# A structural dissection of large protein-protein crystal packing contacts

**DOI:** 10.1038/srep14214

**Published:** 2015-09-15

**Authors:** Jiesi Luo, Zhongyu Liu, Yanzhi Guo, Menglong Li

**Affiliations:** 1 College of Chemistry, Sichuan University, Chengdu, Sichuan 610064, PR China

## Abstract

With the rapid increase in crystal structures of protein-protein complexes deposited in the Protein Data Bank (PDB), more and more crystal contacts have been shown to have similar or even larger interface areas than biological interfaces. However, little attention has been paid to these large crystal packing contacts and their structural principles remain unknown. To address this issue, we used a comparative feature analysis to analyze the geometric and physicochemical properties of large crystal packing contacts by comparing two types of specific protein-protein interactions (PPIs), weak transient complexes and permanent homodimers. Our results show that although large crystal packing contacts have a similar interface area and contact size as permanent homodimers, they tend to be more planar, loosely packed and less hydrophobic than permanent homodimers and cannot form a central core region that is fully buried during interaction. However, the properties of large crystal packing contacts, except for the interface area and contact size, more closely resemble those of weak transient complexes. The large overlap between biological and large crystal packing contacts indicates that interface properties are not efficient indicators for classification of biological interfaces from large crystal packing contacts and finding other specific features urgently needed.

X-ray crystallography is the most popular technique to provide the atomic structure of many protein-protein complexes, and yields the most detailed structural information about the interaction. However, a major problem with this method is that not all interactions observed in the structural data are biologically relevant. Many interactions are artifacts of crystallization that would not exist in the physiological state or in solution. These interactions are called crystal packing contacts or non-specific contacts, as they are not biologically associated. Distinguishing biologically relevant contacts from crystal packing contacts is still a fundamental problem in structural biology^1^,^2^,^3^,^4^.

In recent years, several studies have explored the general interface properties of biological and crystal contacts^3^,^5^,^6^,^7^,^8^. Comparisons between these interactions highlight factors that influence the formation of protein-protein interactions. It has been shown that crystal packing contacts have interfaces that are smaller in size than biological interfaces and have amino acid compositions that are indistinguishable from the rest of the protein surface. These crystal packing contacts also tend to be loosely packed with no definite ‘core’ or ‘rim’ region in the interface because they are less hydrophobic and contain fewer fully buried atoms. In addition, crystal packing contacts also tend to be water rich and less conserved than biological interfaces.

Based on differences in interface properties, a number of computational methods have been developed for distinguishing biological and crystal contacts. In 1998, the PQS used an interface size cut-off of 400 Å^2^ as the main determinant to automatically distinguish between potential quaternary structures as crystal packing or biological oligomers^9^. In 2003, Ponstingl *et al.* proposed a method called PITA for an assembly inference of structures that are likely to be biologically relevant using the properties of contact size and chemical complementarity^10^. In 2005, Liu *et al.* used a combination of four parameters: contact size, packing density, geometric complementarity and potential of mean force to distinguish biological from crystal packing contacts for protein homodimers and monomers^11^. In 2006, Zhu *et al.* integrated six interface properties and implemented them as NOXclass to discriminate between obligate, non-obligate and crystal packing interactions^4^. Tsuchiya *et al.* constructed a server, called PreBI, to predict biological interfaces in protein crystal structures according to the complementarities of the electrostatic potential, hydrophobicity and interface area^12^. In 2007, Krissinel and Henrick developed the PISA method for automatic detection of macromolecular assemblies in the PDB based upon physicochemical models of macromolecular interactions and chemical thermodynamics^2^. In 2008, Bernauer *et al.* presented DiMoVo, a method based on Voronoi tessellation, to discriminate the interfaces between homodimers and crystal packing for a SVM procedure^1^. In 2010, Liu and Li introduced a method called OringPV, which used the propensity vector of residue contacts within the O-ring to distinguish between crystal packing and biological interactions^13^. In 2011, Guharoy *et al.* presented a web server (PRICE) for the analysis of protein-protein interfaces by calculating the degree of conservation as well as the change in free energy binding^14^. In 2012, Capitani’s group made a web server, called EPPIC, to classify small biological interfaces from large crystal contacts by a combination of geometric measure and two evolutionary features^15^. Recently, the EPPIC software has also been run on the entire PDB to analyze oligomerization interfaces in transmembrane proteins^16^ and protein-protein contacts^17^. In 2013, Liu *et al.* proposed a new definition of atomic contacts named β contacts in atomic pair representation for interfaces, and then used them as an SVM input to separate homodimers from crystal packing^18^. Additionally, the β contacts method has successfully been expanded to predict the binding affinity of protein-ligand complexes^19^ and binding hot spots^20^. In 2014, Liu *et al.* also proposed three features related to B factor for the classification between biological interfaces and crystal packing contacts^21^. Their B factor features have shown better performance than the widely used interface size and two published methods, PISA and EPPIC.

Despite the extensive effort made to develop computational methods, distinguishing biological interfaces from crystal contacts remains a challenging task. There are some fundamental difficulties in differentiating biological contacts from crystal ones. First, both contacts have the same physical formation basis. A biological interface could co-exist with 6 ~ 12 different crystal contacts^5^,^22^,^23^. Second, great overlap exists between biological and crystal packing contacts for the interface property distributions. Although some properties such as surface complementarity, hydrophobicity or packing density show clear trends, each alone is insufficient to fully distinguish them as one form of contact^24^. Finally, an important issue is the interface area distribution interval of the crystal contacts employed for training or benchmarking the computational methods^15^,^25^. In many computational methods, the interface area is considered to be the most important feature, and a classifier based on this feature alone can achieve high accuracy^3^ because the area of crystal interfaces is much smaller than that of biological interfaces in their reference datasets. However, the largest interface is not always the biological, more and more crystal contacts are being shown to have similar or even larger interface areas than biological interfaces based on the increasing number of solved crystal structures. Therefore, the performance of methods based mainly on interface area is biased if the large crystal packing contacts are not included.

Recently, little attention has been paid to these large crystal packing contacts, and their structural principles are still unknown. To address this issue, we carried out a comparative analysis of interface properties, focusing on the large crystal packing contacts with an interface area higher than 900 Å^2^, which is very close to the size of biological interfaces. To achieve objective and unbiased analysis results, a large dataset of 773 monomeric protein crystal structures was collected from PDB. Although this number is much smaller than the number of representative protein structures deposited in the PDB, it is currently the largest non-redundant dataset available. Additionally, we classified the crystal packing contacts into two types: general crystal packing contacts and large crystal packing contacts according to the interface area cutoff of 900 Å^2^. To the best of our knowledge, no previous attempts have been made to explore the geometric or physicochemical properties of large crystal packing contacts. This work is the first attempt to dissect the structural principles of large protein-protein crystal packing contacts. Through functional characterization of large crystal packing contacts, we can obtain a better understanding about how specific interactions differ from nonspecific interactions.

## Results

### Structural properties of the interfaces

Table 1 gives the average values of fundamental properties of different types of interfaces.

#### Size of the interfaces

**Figure 1:**
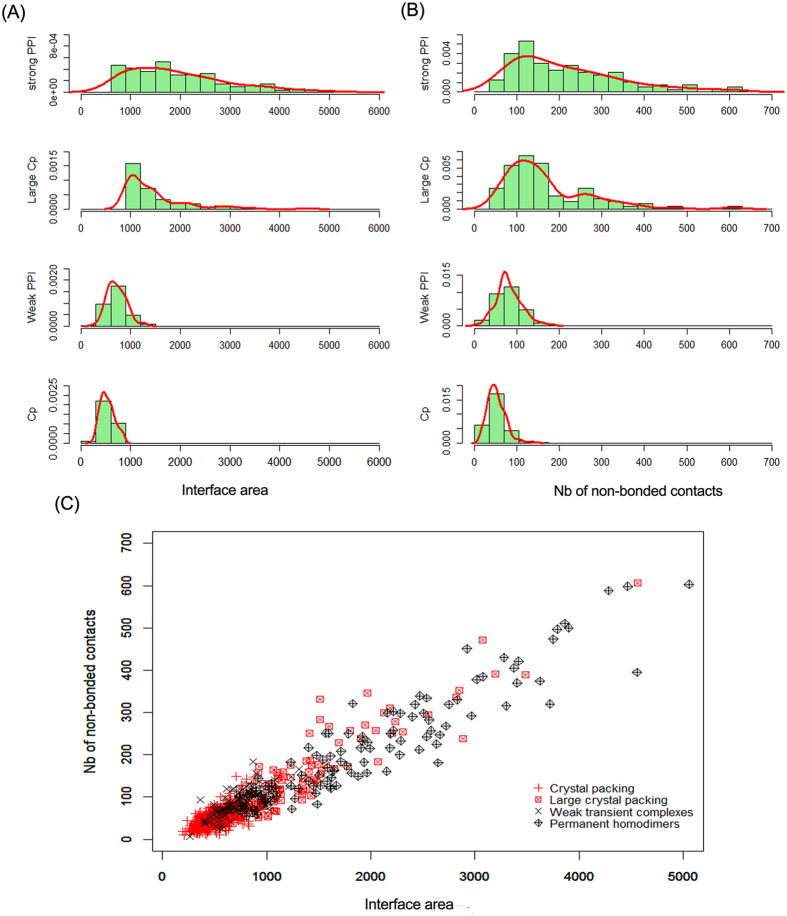
The size properties of protein-protein interfaces: general crystal packing contacts (Cp), large crystal packing contacts (Large Cp), weak transient complexes (Weak PPI) and permanent homodimers (Strong PPI). (**A**) Histograms of the interface area; (**B**) Histograms of number of non-bonded contacts; (**C**) Number of non-bonded contacts and interface area in the four types of interfaces.

The size of the protein-protein interfaces can be quantified by calculating the interface area (IA). The average IA of specific and nonspecific interfaces is listed in Table 1. On average, nonspecific interfaces are smaller in size compared to specific interfaces except for large crystal packing contacts. Permanent homodimers are, on average, approximately 3-fold, 1.3-fold and 4-fold larger than weak transient complexes, large crystal packing interfaces and general crystal packing interfaces, respectively. The weak transient complexes bury an IA in the range of 600–1000 Å^2^, which is considered to be the “standard size” of interfaces occurring in protease-inhibitor and antigen-antibody complexes according to Lo Conte *et al.*^26^. The average IA of crystal packing contacts in our study is 643 Å^2^, which is different from that of previous studies. Janin and Rodier had an average IA of 285 Å^2^, which was obtained from a subset of 1320 crystal packing contacts found in 152 monomeric proteins^5^. Bahadur *et al.* had a larger average IA of 743 Å^2^ that was obtained from a sample of 188 unique pair-wise crystal packing interfaces^7^. However, both data sets focused on different types of crystal packing interfaces. Janin and Rodier calculated all possible crystal packing interfaces in the 152 monomeric proteins. In general, each protein molecule makes 6–12 such crystal packing interfaces and other packing interfaces in the monomeric protein are much smaller than the largest crystal packing interfaces^5^. Bahadur *et al.* only considered the packing interfaces with an IA value of ≥400 Å^2^ and neglected the small crystal packing interfaces. To obtain a comprehensive understanding of the size of crystal packing contacts, we analyzed the packing interfaces in our 773 monomeric proteins. As illustrated in Fig. 1A, the distribution of IA for crystal contacts is quite broad, and therefore, the distributions between specific and crystal packing interfaces significantly overlap with each other. The IA of crystal contacts varied widely from as small as 196 Å^2^ to as large as 4563 Å^2^, with an average value of 643 Å^2^, indicating that the interface area alone is not sufficient to distinguish specific contacts from crystal contacts, especially the large crystal packing contacts. Additionally, the analysis also shows that the weak transient complexes may be the lower limit of IA for specific recognition.

#### The number of non-bonded contacts

The distribution of the number of non-bonded contacts (Nnbc) in Fig. 1B has an almost identical trend to that of IA in Fig. 1A. On average, the general crystal packing contacts have the smallest Nnbc of 52, and permanent homodimers have the largest Nnbc of 216. In addition, the Nnbc of weak transient complexes and large crystal packing is 82 and 169, respectively. Thus, there is a clear although scattered relationship between the interface area and non-bonded contacts, with the larger area containing more non-bonded contacts (Fig. 1C). We also calculated the correlation coefficients (*r*) between IA and Nnbc for each type of interface. The general crystal packing and weak transient complexes have an *r*-value of 0.68 and 0.70, respectively, and the large crystal packing and permanent homodimers achieve a high *r*-value of 0.92 and 0.93, respectively, indicating that the number of non-bonded contacts scales linearly with large interface area.

#### Shape of the interfaces

**Figure 2:**
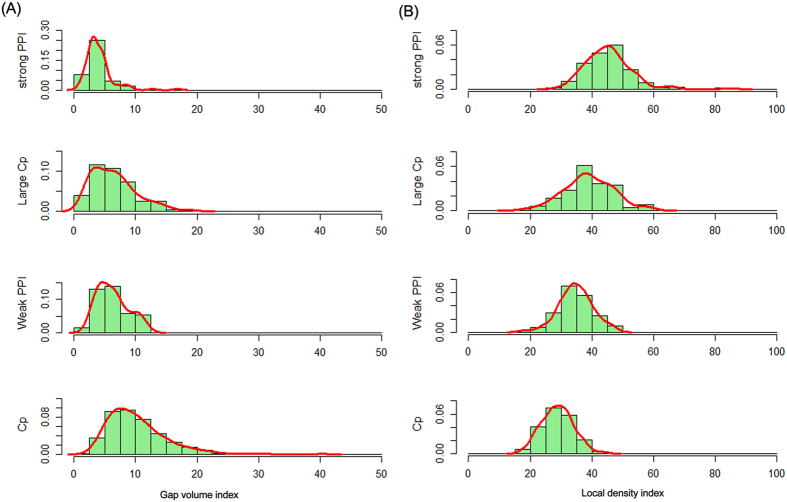
The shape properties of protein-protein interfaces. (**A**) Histograms of gap volume index; (**B**) Histograms of local density index.

The shape complementation or packing density of an interface is an important feature for deciding different types of PPIs. The parameter used in this work to characterize the shape complementation of the interface is the gap volume index (*I*_gap_), which was calculated by relating the volume of the interface cavities to the interface area^27^. Table 1 shows that the general crystal packing interfaces have the largest average *I*_gap_ value, which suggests that they contain a significantly larger cavity volume at their interfaces. However, permanent homodimers have the smallest *I*_gap_, meaning that they are very well packed compared to the other three types of interfaces. However, interfaces of large crystal packing and weak transient complexes are very similar to each other in terms of *I*_gap_ with an average *I*_gap_ of 6 ± 4 Å and 6 ± 3 Å, respectively. Bahadur *et al.*^7^ derived another parameter termed local density index (LD) to measure the packing density at each point of the interface. The LD is the mean number of interface atoms that are within 12 Å of another interface atom, and a high LD value represents a well packed interface. On average, the LD is 29 for general crystal packing, 35 for weak transient complexes, 39 for large crystal packing and 45 for permanent homodimers (Table 1). The distributions of *I*_gap_ and LD for the four types of interfaces in Fig. 2A,B indicate that specific interfaces are more tightly packed than non-specific interfaces and the interfaces of weak PPIs are loosely packed compared to strong PPIs. However, large crystal packing interfaces have a similar shape complementation and packing density as weak transient complexes.

#### Composition of the interfaces

**Figure 3:**
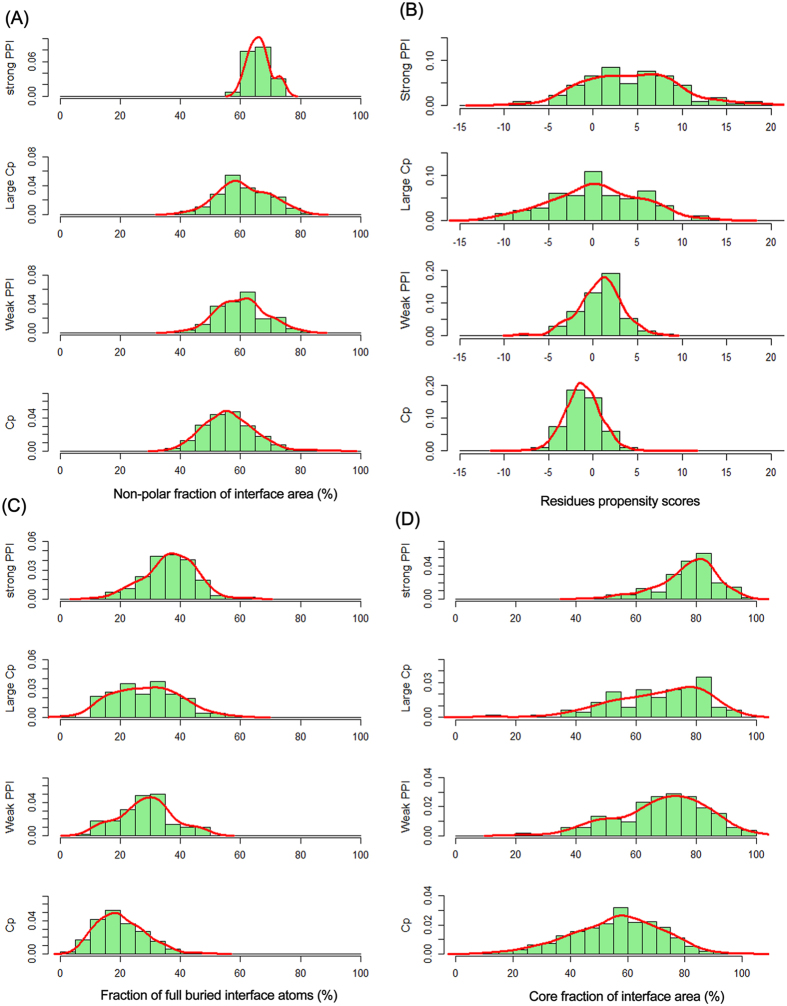
The composition properties of protein-protein interfaces. (**A**) Histograms of non-polar area fraction. (**B**) Histograms of residue propensity score. (**C**) Histograms of fully buried atoms fraction. (**D**) Histograms of core area fraction.

The chemical composition of the interfaces may be divided into two types: non-polar (carbon containing) and polar (N, O and S containing)^24^. As shown in Fig. 3A, the interface area contributed by non-polar atoms varies widely from 36% to 92%, with an average value of 56% in general crystal packing interfaces. As expected, no difference has been found between weak transient complexes and large crystal packing interfaces. Interfaces formed by permanent homodimers tend to be mostly hydrophobic with a narrow distribution and an average value of 66% non-polar. The polar atoms buried at interfaces are expected to form hydrogen bonds^7^,^24^. The comparative analysis of the composition of the interfaces for the four types of interfaces demonstrates that there are on average approximately 10 hydrogen bonds in permanent homodimers and 4 in weak transient complexes. The hydrogen bonds in crystal packing interfaces of monomeric proteins are smaller in number compared to specific interfaces, but the large crystal packing interfaces have approximately 7 hydrogen bonds, which is still very close to that of permanent homodimers (Table 1).

At the residue level, the interfacial compositions of the four types of interactions are different, which can be confirmed by the residues propensity score (*R*_*p*_). A high *R*_*p*_ value denotes that the residues occur more frequently at the interface than on the protein surface. Figure 3B shows that the general crystal packing interfaces have an average *R*_*p*_ close to zero, and specific interfaces have a high positive *R*_*p*_ on average. Large crystal packing interfaces have an average *R*_*p*_ of 0.4, which is more similar to that of weak transient complexes. These results indicate that the amino acid composition of general crystal packing interfaces is very close to that of protein surfaces; however, the specific interfaces are far from the protein surface in distance, and therefore have high *R*_*p*_ values. In large crystal packing interfaces, the composition of the interface residues also differs from that of the protein surface, but the difference is less marked than specific interfaces. In general, specific interfaces are enriched for hydrophobic residues and depleted of charged and polar residues, which is consistent with findings from previous studies^7^,^24^,^25^.

#### Spatial distribution of interface residues

Interface residues also can be divided into core residues and rim residues based on their accessibility to solvent. Core residues have at least one fully buried interface atom (ASA = 0) after complex formation, whereas rim residues contain only accessible atoms 28. Generally, the core residues occupy the interface center and are surrounded by rim residues. The two types of residues have differences in their amino acid composition: the core residues have an amino acid composition similar to that of the protein interior and the rim residues are very similar to the protein surface^29^. In our study, two parameters were used to analyze the spatial distribution of the four types of interfaces. The first is the fully buried atoms fraction (*f*_bu_), which is calculated as the fraction of interface atoms with zero ASA to the total number of interface atoms. On average, approximately 37% of the interface atoms are fully buried for permanent homodimers, this value is only 20% for general crystal packing. Weak transient complexes and large crystal packing have almost the same average *f*_bu_ value of 29% (Table 1 and Fig. 3C). The fully buried atoms may be affected by the packing density of interfaces. Tightly packed interfaces have more contacts and remove water from the core region of the interfaces^25^,^30^.

**Figure 4:**
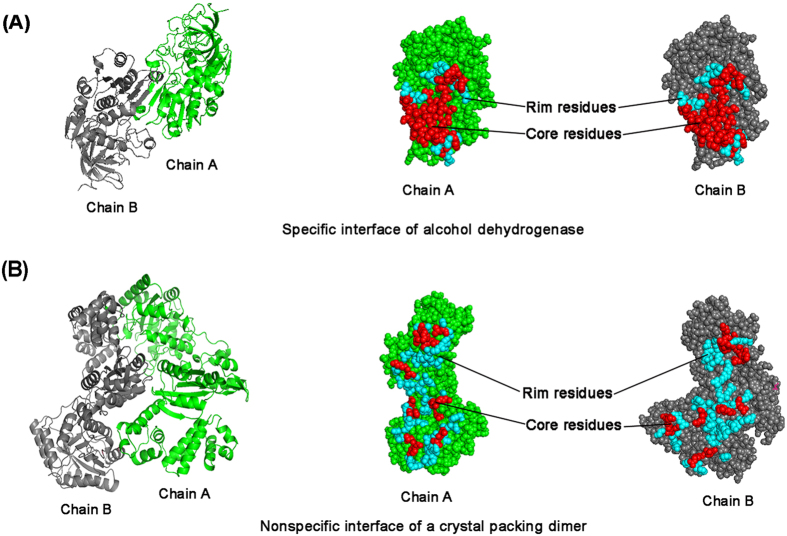
The spatial distribution of interfaces. (**A**) Specific interface of alcohol dehydrogenase (pdb: 2ohx). (**B**) Nonspecific interface of a crystal packing dimer (pdb: 3au2). In both cases, subunits are colored in green and gray, core residues are colored in red and rim residues are colored in cyan, respectively. The figure was created using PyMOL (DeLano Scientific LLC, San Carlos, CA, http://www.delanoscientific.com).

Another parameter is core area fraction (*f*_core_), which is defined as the percentage of the interface area contributed by core residues. On average the core residues constitute 69–77% of the interface area of permanent homodimers and weak transient complexes, but the general crystal packing has a wide distribution, from 9 to 95% with an average value of 56%. However, the large crystal packing interfaces have an almost identical distribution as weak transient complexes (Table 1 and Fig. 3D). The spatial distribution of core and rim residues at specific and nonspecific interfaces is clearly illustrated in Fig. 4, where core residues are colored in red and rim residues are colored in cyan. In crystal packing interfaces of monomeric proteins, core residues are not obvious and are scattered across the interfaces because they are loosely packed and contain very few buried atoms^14^.

#### Segmentation and secondary structure

**Figure 5:**
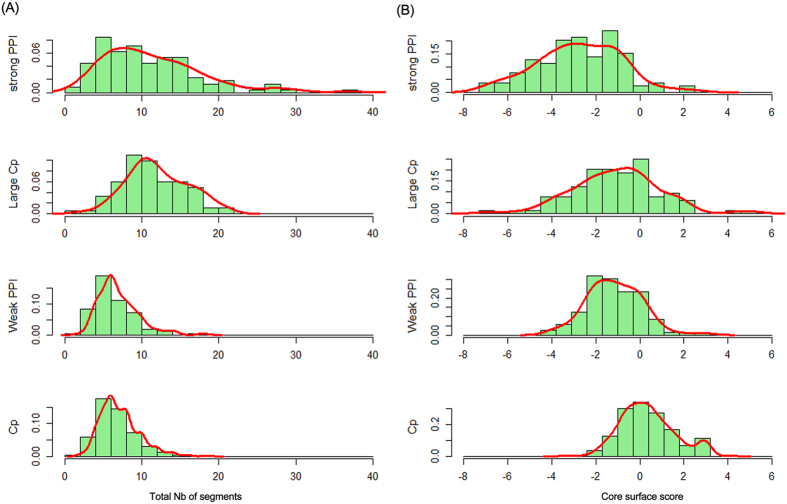
The segmentation and conservation properties protein-protein interfaces. (**A**) Histograms of number of segments. (**B**) Histograms of core-surface score.

The number of discontinuous segments involved at the interface is important because it highlights the possibility of using corresponding peptides as a mimic for the interaction^31^,^32^. To analyze the discontinuous nature of the interfaces, the mean number of segments at the interfaces was calculated for each type of interaction (Table 1). The number of segments varied from 1 to 37 (Fig. 5A). General crystal packing and weak transient complexes have an average of 7 segments, which is only half the value of the large crystal packing and permanent homodimers. Large interfaces, whether specific or non-specific, generally have more peptide segments. However, differences are observed after the number of segments is normalized relative to the length of segment and the size of the interface. Our results show that non-specific interfaces are generally more fragmented than specific interfaces^32^.

**Figure 6:**
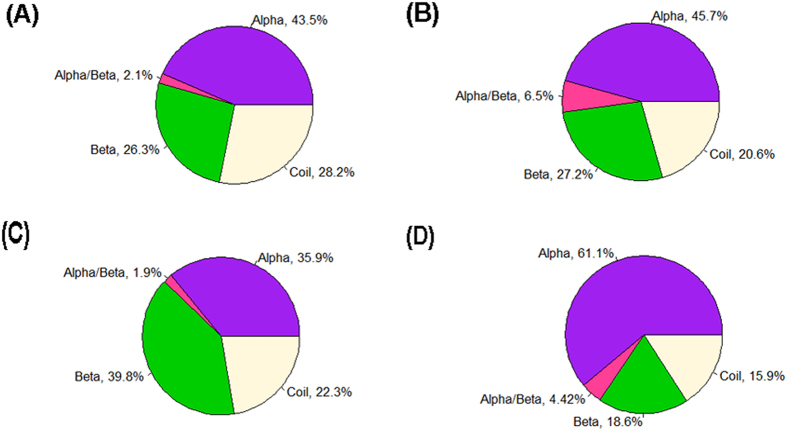
Schematic diagram showing distribution of four categories interfaces. (**A**) General crystal packing contacts. (**B**) Large crystal packing contacts. (**C**) Weak transient complexes. (**D**) Permanent homodimers.

We also analyzed the secondary structure of the interface regions. Four categories of interfaces based on the proportion of secondary structural elements were defined as an alpha interface with an alpha-helix content more than 30% and beta-strands content less than 30% or a beta interface with an alpha-helix content less than 30% and beta-strands content more than 30% or an alpha/beta interface with an alpha-helix content more than 30% and beta-strands content more than 30% or a coil interface with an alpha-helix content less than 30% and beta-strands content less than 30%^25^. The distributions of the four types of interfaces are shown in Fig. 6. The alpha and beta interfaces are more abundant in permanent homodimers and weak transient complexes with a percentage of 61.1% and 39.8%, respectively. Beta interfaces are almost equally abundant in both general crystal packing and large crystal packing. However, more general crystal packing occurs in the coil interface (28.2%) group compared to large crystal packing (20.6%).

#### Core-surface conservation scores

We calculated the core-surface conservation score as proposed by Duate *et al.*^15^. This score provides a measure of the selection pressure acting on the key residues of an interface core region compared to the surface residues. These were defined as the ratio of the average sequence entropies of core residues to those of surface residues. Figure 5B shows that the general crystal packing interfaces have an average core-surface conservation score close to zero and specific interfaces have negative core-surface conservation scores on average. The large crystal packing interfaces have an average score of −1.05, which is more similar to that of weak transient complexes (−1.15), indicating that core residues are more conserved than surface residues in biological contacts. However, there is no significant difference in the conservation of core residues and surface residues in crystal packing contacts. In large crystal packing interfaces, the conservation of the core residues also differs from that of the protein surface, but the difference is less marked than specific interfaces.

### Performance of different computational methods for discriminating specific and non-specific interfaces

A series of breakthroughs in protein production and structure determination techniques, especially in protein crystallography have enabled researchers to solve the structure of macromolecular complexes and investigate the mechanisms of PPI formation. Unfortunately, the experimental determination of PPIs is tedious and difficult. Alternatively, PPIs can be predicted by computational methods. Although less accurate than experimental observations, computational predictions can be sufficiently useful to prompt functional hypotheses and guide experiments. The analysis of different types of PPIs as described above shows that large crystal packing and specific interfaces have similar geometric and physicochemical properties. We conclude that the similar properties between the two types of interfaces would make them more difficult to distinguish from each other. To test the performance of the current computational methods on the large crystal packing contacts, four popular methods were selected including PITA^3^, PISA^2^, DiMoVo^1^ and EPPIC^15^. Table 2 shows that when predicting the interfaces for a set of 92 large crystal packing and 103 weak transient complexes, high error rates are observed in all of the methods. However, it is unfair to make a conclusion that these computational methods fail in the case of large crystal packing or weak transient complexes because they were trained or benchmarked using the general crystal packing and permanent homodimers. We denote that the similar functional features between the large crystal packing and weak transient complexes are often ignored in previous studies. More attention needs to be paid to the large crystal packing interfaces and it is urgently to find and specially characterize new determinants so that the performance of computational methods can be improved.

## Discussion and Conclusion

This study not only systemically analyzed different types of interfaces on a large scale but also greatly expands current knowledge regarding the principles governing protein-protein recognition. The geometric and physicochemical properties of interfaces were analyzed including size, shape, composition, spatial distribution, segmentation and secondary structure. We found that large crystal packing interfaces and specific interfaces show no difference in most of the above properties.

This research also raises an important issue regarding how much the appearance ratio of large crystal packing contacts is observed in protein crystals. Although it is difficult to accurately estimate the coverage of large crystal packing contacts to all crystal packing contacts, a rough estimate of approximately 12% was made by comparing 92 large crystal packing contacts with 773 monomeric protein crystal structures. Such an estimate may not be very accurate. Recently, Baskaran *et al.*^17^ carried out a PDB-wide, evolution-based, classification of protein-protein contacts and obtained a large-scale dataset of crystal contacts called XtalMany, which contain nearly 2913 crystal interfaces. In the XtalMany dataset, 430 interfaces, with an interface area higher than 900 Å^2^ are considered to be large crystal packing contacts. Approximately 15% of crystal packing contacts are large interfaces in the XtalMany dataset and this ratio is similar to our estimation. Although the ratio of large crystal packing contacts seems relatively low only approximately 12 ~ 15%, this proportion does not mean equal small numbers of large crystal packing contacts in the crystal structures. With the growth in crystal structures, more large crystal packing contacts will be found in the future, and it is impossible to ignore their specific features when constructing models for classifying specific and non-specific interfaces.

Another issue we addressed in this study is whether the space groups affect the properties of crystal packing interfaces. Goodsell and Olson have shown that in crystals, the major types of interactions are found where the contacts are related by twofold symmetry. In addition to this twofold symmetry, crystal contacts can also have higher point group symmetry, which is, however, quite rare^33^. Janin and Rodier showed that crystal interfaces that incorporate a twofold symmetry, on average, have larger and probably more stable interfaces than those without this symmetry^5^. In the present study, among the numerous space groups of the crystals, we observed 9 space groups at least 20 times. These space groups are P 1 21 1, C 1 2 1, P 21 21 2, P 21 21 21, C 2 2 21, P 41 21 2, P 43 21 2, P 31 2 1 and P 32 2 1. We found that low symmetry space groups, for example P 1 21 1 (104), have more monomeric protein crystal structures than high symmetry space groups such as P 31 2 1 (27) and P 32 2 1 (34). This finding is inconsistent with the results from Goodsell and Olson. The inconsistent results are mainly caused by the great difference of the data used in our wok and previous studies. In this work, we firstly analyzed the largest number of 773 protein crystal structures and no more than 100 structural were used in other works. In addition, only the monomeric protein structures were investigated in this work and others in the multimeric protein structures, although they are few, were not included. However, the comparison of geometric and physicochemical properties between the 9 space groups showed that there is no difference between them.

The 773 monomeric protein crystal structures also allowed us to analyze how many molecules surround each reference molecule in a crystal. Carugo *et al.*^34^ analyzed these monomeric protein crystal structures and observed that there are as few as 3 or as many as 18 molecules around the reference molecule, with an average number approximately 10. In addition, there are more molecules around reference molecules with low symmetries as compared to the high symmetries, and the number of molecules is independent of the protein shape and dimension.

In conclusion, although crystal packing contacts have been explored for many years, many issues remain unresolved. In this study, we found no significantly different geometric and physicochemical properties in large crystal packing interfaces and specific interfaces. This study represents the first large-scale analysis specifically examining large crystal packing contacts. This work will provide a better understanding of the principles governing protein-protein recognition in the classification of specific and nonspecific interactions and in guiding protein crystallization. However all analysis were implemented on the crystal packing contacts generated in the monomeric protein structures. We know that proteins functions are usually in complexes with higher multiplicities, so with the more accurate PPI contact data in the multimeric protein structures are available in the future, a more comprehensive structural dissection of the large crystal packing contacts can be expected not only in the monomeric protein structures but also the multimeric protein structures.

## Methods

### Data sets

All crystal packing contacts structures were taken from the work of Carugo *et al.*^34^, after a series of data processing steps. PDB entries known to dimerize upon ligand binding, for instance, when binding to RNA or DNA, were not considered in the data set. Since the multimeric protein structures usually contain both biologically relevant contacts and crystal packing contacts, it is still difficult to accurately distinguish them in the multimeric protein structures using currently available methods. The monomeric protein structures are the simplest case of crystal packing contacts, because any contacts in these structures are clearly not biologically associated. In order to avoid introducing data bias, only monomeric proteins that crystallize with one molecule in the asymmetric unit were retained to limit the analysis to crystal packing contacts through exclusion of specific interactions. Furthermore, entries containing only Cα atoms were disregarded and only those structures with crystallographic resolution better than 2.5 Å were retained. To avoid redundancy and homology bias, proteins were aligned by the CD-HIT program^35^ with a sequence identity threshold of 50%. Next, entries with <50 amino acids or containing more than 5% non-protein atoms (excluding water molecules) were screened out, which resulted in a dataset of 773 PDB structures. Hereafter, the molecule in the asymmetric unit is deemed the “reference molecule” and other molecules surrounding it by symmetry operations are the “neighbor molecules”. The intermolecular contacts between the reference molecule and the neighbor molecules are considered to be crystal packing contacts. All crystal packing contacts were generated using EPPIC program^15^ and two molecules that exhibited the largest interface area were retained in each entry. In addition, 92 entries that bury more than 900 Å^2^ of interface area were selected from the 773 PDB structures as a large crystal packing data set. It is important to recognize that, so far, there is no clear definition of large crystal packing contacts, and from the above analysis, it can be observed that a continuum distribution of interface area does exist in the crystal packing interfaces, and these significantly overlap with specific interactions. Reviewing previous studies^26^,^36^, antigen-antibody, protease-inhibitor and most heterodimeric complexes bury an interface area in the range of 600–1000 Å^2^ that is labeled a ‘standard size’ interface and an area above the ‘standard size’ could be considered a large interfaces. We chose an interface area cutoff of 900 Å^2^ for large crystal packing contacts to avoid overlapping with ‘standard size’ specific interactions. The interface area may influence other properties of protein-protein interfaces, such as shape, hydrophobicity or residue interface propensities. Any comparison between geometric and physicochemical properties of different interface types needs to take into account the impact of interface area. For this reason, we implemented an additional control experiment in our study. We compiled another dataset in which the interface area difference between each strong protein-protein interaction and its corresponding large crystal packing or between each weak protein-protein interaction and its corresponding crystal packing is less than 10 Å^2^ to eliminate the effect of a large interface area difference. Then, the same feature analysis on this new dataset was performed, and the results are shown in Supplementary Figures 1. The trends in Supplementary Figures 1 are almost identical to those in Figs 1, 2, 3 and 5, suggesting that the distributions of different types of protein-protein interactions are strictly comparable when comparing their geometric and physicochemical properties.

Specific interactions can be discriminated as permanent interactions and transient interactions based on the lifetime or stability of the complex^37^,^38^,^39^. Most homodimers are permanent as they assemble tightly as soon as they are synthesized and can stay together longer than the life of a cell. Based on the work of Bahadur *et al.*^29^, 117 homodimers with permanent or strong interactions were retrieved. Transient protein-protein complexes are generally considered weak or non-obligate interactions^4^,^7^. However, according to the definition of Perkins *et al.*^39^, transient interactions can be further subdivided into weak and strong. Weak transient interactions have a fast bound-unbound equilibrium with a dissociation constant (*K*_d_) value typically in the μM range. Strong transient interactions, triggered by the binding of an effector molecule or a conformational change, may last longer and have a continuum of *K*_d_ that exists between weak and permanent interactions^37^,^38^,^39^. In our study, we used 103 weak transient complexes with *K*_d_ values higher than 1.0 × 10^−6^ M as weak interactions from our previous work^30^.

### Definition of interface properties

We calculated nine important interface properties to reveal the structural basis of different types of PPIs. They are the interface area (IA), number of non-bonded contacts (Nnbc), number of segments (Ns), core area fraction (*f*_core_), non-polar area fraction (*f*_np_), fully buried atom fraction (*f*_bu_), residue propensity score (*R*_p_), local density index (LD) and gap volume index (*I*_gap_). A residue is defined as an interface residue if its solvent accessible surface area (ASA) decreases by >1 Å^2^ upon binding^31^. We calculated ASA using the NACCESS program (Hubbard, S. J. & Thornton, J. M. NACCESS, computer program. *London: Department of Biochemistry and Molecular Biology, University College London.* 1993.), with a probe sphere of radius 1.4 Å. Residues with one or more completely buried interface atoms (ASA = 0) are considered to be core residue^29^. Two atoms are considered to be non-bonded contact across the interface if they are at a distance smaller than 3.9 Å, which is a default distance calculated by the 2P2I inspector tool (http://2p2idb.cnrs-mrs.fr/2p2i_inspector.html). An interface segment is defined as a stretch of residues that starts and end with interface residues and may contain intervening non-interface residues, but only in stretches of not more than four^32^.

#### Interface area

IA is defined as one half of the total decrease of ASA of two proteins A and B upon interaction and it reflects the size of the interface:


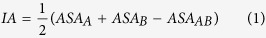


#### Fully buried atoms fraction

The *f*_bu_ is defined as:


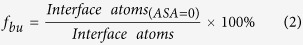


where the *Interface atoms*_*(ASA=0)*_ represents the interface atoms fully buried in the complex with zero ASA.

#### Non-polar area fraction

At a high resolution, the chemical groups at the protein surface may be divided into two types: non-polar (carbon containing) and polar (N, O, and S containing). The *f*_np_ reflects the hydrophobic property of the interface and is defined as:


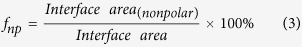


where the *Interface area*_*(nonpolar)*_ represents the interface area contributed by non-polar (carbon containing) interface atoms.

#### Core area fraction

Interface residues can be divided into the core residues and the rim residues based upon their accessibility to solvent. The core residues have at least one fully buried interface atom (ASA = 0) after complex formation, whereas rim residues are those having accessible atoms only^40^. Generally, the core residues occupy the interface center and are surrounded by rim residues. The *f*_core_ reflects the size of the interface core region and is defined as:


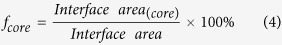


where the *Interface area*_*(core)*_ represents the interface area contributed by core residues.

#### Residue propensity score

According to the description given by Bahadur *et al.*^7^, the selection or exclusion of a certain type of amino acids at an interface can be expressed as a set of propensities:


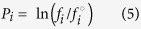


where *f*_*i*_ is the number or area fraction contributed by residue type *i* to the interface, and *f*_i_^о^ is the equivalent fraction contributed to the protein surface. Summing the propensities of all the residues present in an interface yields the *R*_*p*_ score. A high *R*_*p*_ value denotes that the residues occur more frequently in the interface than on the protein surface.

#### Local density index and gap volume index

The shape of the interfaces is measured by the gap volume index and local density index. The local density index is defined as the work by Bahadur *et al.*^7^. For each interface atom *a*, the number *n*_*a*_ of interface atoms are counted within an optimized distance of 12 Å of atom *a* in the same subunit and then *n*_*a*_ is averaged over all *N* interface atoms:


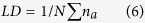


The larger the local density index, the more complementary the interface shapes are. The gap volume index is computed by normalizing the gap volume of the interface with its interface area^41^.


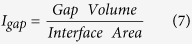


The smaller the gap volume index, the more complementary the interface shapes are. Gap volume between the two molecular surfaces was computed using the program SURFNET. In this work, all interface properties were calculated using the ProFace program (http://resources.boseinst.ernet.in/resources/bioinfo/interface/) and 2P2I.

#### Core-surface conservation scores

Duate *et al.*^15^ used the following procedure to calculate sequence entropies for each residue of a given PDB structure. First, they found the reference UniProt sequence for the PDB sequence and searched the UniRef100 database through BLAST software with soft cut-off of 60% identity, 80% coverage filters and a maximum number of 100 to find putative homologs of the reference UniProt sequence. Then, they used BLASTCLUST to cluster the sequences and a single representative from each cluster was chosen. Finally, they used the CLUSTALO program^40^ to perform a multiple sequence alignment of the selected homologs and calculated the sequence entropies using the following equation based on these sequence alignments:


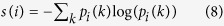


where 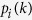 is the probability of a residue of class *k* that is found at position *i* of the alignment and the 20 amino acid types are grouped in ten classes as proposed by Murphy *et al.*^42^. Entropy values were finally mapped back to the PDB sequences to compute the core-surface score.

### Performance assessment

The prediction performance was assessed by four measures: sensitivity (Sn), specificity (Sp), accuracy (Acc) and Matthews Correlation Coefficient (MCC). They are defined as follows:


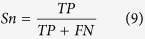



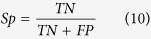



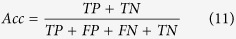



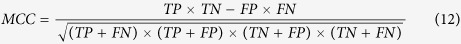


where TP, TN, FP and FN represent the true biological interface, the true crystal packing contact, the false biological interface and the false crystal packing contact, respectively.

## Additional Information

**How to cite this article**: Luo, J. *et al.* A structural dissection of large protein-protein crystal packing contacts. *Sci. Rep.* **5**, 14214; doi: 10.1038/srep14214 (2015).

## Supplementary Material

Supplementary Information

## Figures and Tables

**Table 1:** Average geometric and physical-chemical properties of different types of PPIs.

Interface	**Non-specific protein-protein interaction**		**Specific protein-protein interaction**	
	**General crystal packing contacts**	**Large crystal packing contacts**	**Weak transient complexes**	**Permanent homodimers**
Number	681	92	103	113
Nb of interface residues	35 ± 10	84 ± 32	42 ± 12	104 ± 50
Nb of interface atoms	115 ± 31	306 ± 133	151 ± 42	400 ± 198
Nb of non-bonded contacts	52 ± 22**	169 ± 99	82 ± 31**	216 ± 127*
Nb of segments	7 ± 3**	12 ± 4	7 ± 3**	12 ± 6
Nb of hydrogen bonds	2 ± 2	7 ± 6	4 ± 3	10 ± 8
Interface area (Å^2^)	531 ± 147**	1472 ± 661	718 ± 195**	1950 ± 986*
Interface area ratio (%)	6 ± 3	11 ± 6	9 ± 4	16 ± 7
Non-polar area fraction (%)	56 ± 9*	61 ± 8	60 ± 8	66 ± 4*
Core area fraction (%)	56 ± 16**	67 ± 16	69 ± 15	77 ± 10*
Fully buried atoms fraction (%)	20 ± 8**	29 ± 11	29 ± 9	37 ± 9*
Residue propensity score	−1.1 ± 2.1*	0.4 ± 5.0	0.8 ± 2.5	4.3 ± 5.2*
Local density index	29 ± 5**	39 ± 8	35 ± 6*	45 ± 8*
Gap volume index	10 ± 5**	6 ± 4	6 ± 3	4 ± 2*

Data are expressed as mean ± SD.

Asterisks mark the statistical significance of the differences between large crystal packing contacts and the other types of interaction as follows: *p < 0.05 and ** *p *< 0.001.

**Table 2:** The performance of different computational methods on the 92 large crystal packing and 103 weak transient complexes.

	**Sn (%)**	**Sp (%)**	**Acc (%)**	**Mcc**
PITA^a^	38.3	46.7	42.6	−0.14
PISA^b^	19.4	48.9	33.3	−0.33
DiMoVo^c^	33.0	60.0	45.6	−0.07
Eppic	49.5	51.1	50.2	0.006

^a^A PITA score above 70 indicates a specific interface and a score below 70 is taken to identify a nonspecific interface.

^b^The ΔG of PISA indicates the solvation free energy gain upon formation of the interface in kcal/M^2^. A value below −10 kcal/M is considered to be a specific interface otherwise it is a nonspecific interface.

^c^A DiMoVo score above 0.5 indicates a potential biological complex, and a score below 0.5 indicates a crystal dimer.
