# Supplementary material for: A structural dissection of large protein-protein crystal packing contacts
Source: Sci Rep. 2015 Sep 15;5:14214. doi: 10.1038/srep14214 (PMC4572935; doi:10.1038/srep14214)
Supplement: Supplementary Information [file srep14214-s1.pdf]

Supporting Information

A structural dissection of large protein-protein crystal packing contacts

Jiesi Luo, Zhongyu Liu, Yanzhi Guo*, Menglong Li*

College of Chemistry and State Key Laboratory of Biotherapy, Sichuan University, Chengdu, Sichuan 610064, PR China

Corresponding authors marked with *

Yanzhi Guo

Menglong Li

Tel: +86 28 85413330

Fax: +86 28 85412356

E-mail address: yzguo@scu.edu.cn

liml@scu.edu.cn

IA: Interface area
NA: Number of atoms
NR: Number of residues
fnp: Non-polar interface area
fbu:Fully buried atoms fraction
fcore:Core area fraction
Nnbc:Nb of non-bonded contacts
Nbseg:Total Nb of segments
HB:Hydrogen bonds
SSI:Secondary structure at interface
Igap:gap index

general crystal packing contacts

		IA	NA	NR	fnp	fbu	Rp	LD	Nbseg	Nnbc	HB	Igap	SSI	fcore
1wvr	P 43 21 2	898.89	188	56	47.56088	15	-4.62	28.4	11	68	1	8.6394	alpha	44.36583
1pii	P 41	892.755	188	49	57.00276	25	-0.62	34.91	8	69	1	7.050501	alpha	63.15114
3m6c	P 21 21 2	886.965	164	50	75.53906	30	2.58	39.095	6	78	3	3.641485	alpha	72.00228
3cjj	P 21 21 2	884.68	178	50	70.38816	35	0.36	35.35	10	78	6	3.902677	beta	85.18673
2oit	P 21 21 21	884.635	183	47	54.44279	31	-1.67	40.305	8	148	4	6.946594	coil	79.92562
1ex7	P 43 21 2	882.6	202	50	63.18491	25	-1.86	32.87	6	68	2	6.515976	alpha	63.38319
2gko	P 41 21 2	881.57	198	58	58.85863	16	2.56	34.95	12	84	4	9.992116	coil	50.19114
1k0f	C 1 2 1	870.21	196	64	47.12081	18	-2.32	35.2	12	133	0	5.511164	alpha	38.48152
2zzr	P 32 2 1	868.84	184	56	56.52019	26	1.3	35.85	8	113	0	12.85766	alpha	49.71456
1ci3	H 3 2	867.67	202	62	58.34476	15	-1.24	30.48	14	84	2	13.91745	alpha	59.15037
1fe5	R 3 2	865.5	173	54	56.59041	9	0.36	36.4	8	80	1	4.940635	coil	29.42808
3b4x	C 1 2 1	865.065	183	48	63.88537	23	-2.61	36.495	5	75	4	9.714588	alpha	63.56459
3m70	C 2 2 21	864.85	166	62	55.34254	7	-3.52	20.87	18	80	8	11.76967	beta	16.95554
2d5b	P 21 21 21	861.675	186	59	44.38797	9	-8.14	20.345	16	77	9	16.22334	alpha	28.85427
3prt	P 63 2 2	861.24	202	58	47.59997	47	-3.2	44.28	8	131	2	4.055919	alpha	83.28224
3k1d	P 21 21 2	860.19	201	62	58.37431	12	-0.13	23.16	19	69	0	18.28767	coil	47.74003
2cw4	H 3	854.495	191	54	74.52355	38	3.82	39.075	8	112	5	4.012897	beta	79.99696
1ako	P 31 2 1	849.35	172	48	56.1135	23	-1.84	33.28	8	90	5	7.053182	alpha/Bet	49.76158
1ppo	P 31 1 2	844.09	182	52	52.6105	27	-0.04	30.57	12	107	2	4.690104	coil	81.48065
1mrj	P 21 21 21	840.535	172	55	45.07665	23	-1.44	29.73	10	105	5	8.014538	alpha	52.31727
1f6w	P 21 21 21	831.485	191	59	61.80448	16	-3.71	29.28	12	32	1	9.883666	alpha	42.44454
2p8v	P 32 2 1	828.88	183	47	62.79075	27	0.51	40.485	7	103	4	4.246839	beta	75.98989
3dha	P 21 21 21	827.535	167	45	53.4636	22	0.06	32.67	9	95	7	9.066227	beta	67.41165
2ztg	C 1 2 1	826.995	200	63	50.03718	9	-10.12	21.105	14	43	0	19.76856	coil	37.35815
1n9l	P 65 2 2	826.48	156	46	78.70608	36	3.02	30.56	6	54	0	5.067721	beta	64.01244
3elo	P 63	825.76	136	36	48.41177	23	-1.65	35.025	8	81	5	5.374588	alpha	53.2128
1a8q	P 63	825.2	186	51	69.33471	34	-0.09	39.725	9	76	0	5.64817	alpha	72.83568
1crl	C 2 2 21	823.54	134	50	89.81592	12	10.3	18.3	12	37	2	15.45827	alpha	25.95745
3ecm	C 2 2 21	822.3	182	52	50.14228	24	2.22	35.67	6	76	6	7.461693	alpha	57.1227
2okt	P 1 21 1	820.335	182	52	54.37778	18	0.11	32.715	10	75	4	9.376194	coil	56.15998
1u6t	P 21 21 21	818.645	177	57	57.25009	14	-3.14	27.635	10	71	3	8.797769	alpha	48.5644
1qgi	P 21 21 2	818.545	166	50	47.53862	16	-3.59	30.835	9	67	3	10.3739	alpha	51.99164
2q88	P 43 21 2	815.49	158	64	50.78542	5	-3.06	22.68	10	80	2	10.80592	beta	13.11359
2j9v	C 2 2 21	812.24	168	44	69.46592	48	3.76	42.36	4	106	4	4.483429	alpha	86.80439
1h6t	P 21 21 21	811.445	189	55	56.57253	26	-5.36	31.925	12	117	2	14.58232	alpha	67.68296
2iwc	P 41 21 2	806.075	180	49	51.22166	27	-3.15	33.85	8	70	4	6.012468	alpha	67.92296
2aiq	C 1 2 1	806.06	176	48	58.39764	48	2.14	43	10	143	4	7.063519	alpha	90.88778
1tyj	I 2 2 2	804.98	170	52	47.54528	19	5.14	29.36	12	81	5	5.660079	beta	51.03232
2v3q	C 2 2 21	802.29	154	56	57.8706	14	-0.84	28.03	14	65	2	11.1688	coil	29.21263
2bvl	P 21 21 21	799.62	159	47	51.89965	24	-0.92	26.31	7	91	2	12.82687	alpha	68.56007
1qaz	C 1 2 1	799.54	168	50	67.18738	12	-2.58	22.24	14	56	2	11.38874	coil	43.79643
1ks8	C 1 2 1	797.35	178	50	49.80749	20	2.26	30.13	14	110	0	9.159717	alpha	65.57221
3ewk	P 4 21 2	796.695	173	48	59.37216	19	1.57	31.265	8	82	1	7.646427	alpha/bet	46.95398
3d3y	I 4	796.495	196	57	58.0192	17	-3.12	31.35	11	70	5	13.7628	coil	59.90747
2r0s	H 3 2	790.315	170	54	55.23367	12	-1.3	38.82	8	69	2	12.73874	alpha	48.68312
1a58	P 41 21 2	789.695	172	54	60.54806	26	1.87	36.045	10	56	0	6.410703	beta	61.93974
3a7r	P 61 2 2	787.49	188	70	55.9042	15	-0.88	26.16	16	76	5	11.77729	alpha	47.28441
2jxr	P 32 2 1	784.27	164	50	70.53821	12	3.64	28.49	10	43	0	11.48998	coil	10.61369
3mwq	C 1 2 1	781.8	168	56	61.82144	21	-4.88	30.69	10	72	6	5.063789	beta	59.47557
2wc1	P 41 21 2	776.85	156	42	67.43387	26	-1.4	37.31	8	108	6	5.061299	alpha	56.08161
3buj	P 41 21 2	775.14	170	60	39.06133	14	-6.56	33.76	12	88	2	8.869198	alpha	47.1102
2osn	R 3 2	774.585	167	54	52.35836	17	1.36	32.815	8	60	2	6.073898	coil	54.46336
3pen	I 2 3	774.575	168	43	58.20676	18	-1.94	35.615	9	67	3	7.433431	beta	52.0337
3n35	P 31 2 1	772.3	142	46	67.79749	28	0.02	34.31	8	36	0	5.646122	beta	71.39324
3hxw	P 41 21 2	771.705	163	52	51.07198	23	-1.98	30.18	11	61	6	9.547197	alpha/bet	66.73016
1xw3	P 32 2 1	770.95	176	42	62.31014	27	-0.2	31.82	6	127	6	3.375212	beta	78.41365
3fvz	P 21 21 21	767.715	167	46	65.75357	38	1.5	41.035	10	110	3	7.258058	beta	78.83003
1hd2	P 41 21 2	760.45	158	42	74.52166	38	2.58	38.08	10	52	2	4.136367	alpha	71.71017
1yg9	C 1 2 1	757.95	142	44	60.03298	14	0.12	19.92	12	128	4	14.28458	coil	44.89082
2fph	P 1 21 1	756.345	163	51	61.27429	12	-0.69	26.685	10	47	1	8.304239	alpha	41.04939
2ej9	C 1 2 1	753.305	152	39	56.69815	19	0.47	36.515	8	67	3	5.649597	alpha/bet	62.34128
3pdd	P 21 21 21	750.28	151	46	56.09439	20	-2.21	26.925	9	85	6	6.419097	coil	57.70846
2gke	C 2 2 21	749.2	164	42	56.13187	43	2.56	38.98	8	86	5	3.486719	beta	88.1874

1inp	P 41	748.115	168	50	46.02501	25	-3.86	31.775	10	81	3	11.90542	coil	69.62499
1xwl	P 21 21 21	747.16	181	52	46.16883	22	-3.73	28.76	13	80	2	11.46441	alpha	60.27357
1akl	P 21 21 21	745.25	164	49	54.34821	29	-0.95	36.83	9	96	4	11.00922	coil	56.19658
1c44	P 41 21 2	744.205	154	44	69.80335	44	-0.99	42.76	5	82	0	4.535041	alpha	70.52963
1h3q	C 2 2 21	743.58	150	44	55.73442	5	-2.78	29.25	4	49	0	8.31971	alpha	43.11439
1qsa	P 21 21 21	741.995	160	49	44.50636	17	-1.85	23.67	11	76	4	15.76071	alpha	52.19375
3ql6	P 1 21 1	741.695	150	47	60.02198	11	-2.21	18.63	14	47	1	15.62603	coil	36.37749
1hg8	P 21 21 21	741.13	162	59	44.80658	11	-2.87	26.74	16	62	3	14.18526	coil	33.71743
2xsp	C 1 2 1	740.01	144	46	46.08451	8	-1.86	32.17	10	70	0	13.26266	alpha	25.45236
1zx5	P 43 3 2	738.17	158	46	43.23462	22	-4.9	24.91	14	97	4	10.56616	coil	58.38899
1vff	C 1 2 1	732.16	164	46	54.79267	29	-1.9	36.85	4	46	0	7.86406	alpha	73.87183
2z5y	C 2 2 2	731.1	154	36	79.3202	21	3.82	31.74	2	45	0	8.600219	alpha	63.71905
1roc	C 1 2 1	729.675	152	46	55.15195	19	-0.87	31.49	7	85	2	8.390379	beta	66.82701
1qho	P 31 2 1	729.07	166	64	50.08435	16	-3.74	22.31	14	68	6	23.02093	coil	38.80834
1dq3	P 1 21 1	728.05	160	40	52.61864	26	0.03	32.76	8	62	3	5.618433	beta	55.9213
2dt8	C 1 2 1	728.04	176	42	57.59848	23	-4.32	37.3	4	74	0	5.93374	alpha	48.43827
3ooi	P 21 21 21	724.68	149	37	62.55934	23	0.61	34.505	7	51	2	7.889344	alpha	61.84868
3ils	P 21 21 21	724.55	149	45	66.07757	15	3.08	30.975	10	54	0	9.451204	beta	59.1857
2i6v	P 21 21 21	722.655	156	40	66.68742	25	2.9	37.985	5	68	3	4.791706	alpha	79.30479
2cc1	P 21 21 21	720.165	160	48	52.58309	34	-1.3	35.855	7	87	4	8.482431	alpha	74.31144
1ifg	P 43 21 2	719.73	130	42	48.43344	14	-3.42	32.03	10	47	5	11.56839	beta	39.44813
1x8q	C 1 2 1	719.23	146	50	56.61888	33	0.3	33.37	8	76	4	7.156084	beta	76.87666
2jfr	P 21 21 2	718.64	140	49	48.82556	12	-1.49	30.3	7	90	7	10.81105	coil	44.46802
1bwk	P 43 21 2	718.56	152	52	70.11384	28	-2.5	32.53	8	60	2	6.209294	alpha	57.78223
2vhk	P 41 21 2	713.12	142	40	63.45356	17	-0.62	29.66	10	84	0	8.56149	beta	41.82886
2x49	P 21 21 21	713.025	150	43	42.89331	14	-1.85	28.915	10	62	4	11.58251	coil	52.69731
2q8w	I 2 2 2	712.5	164	44	48.81825	27	-0.84	37.17	7	69	7	5.480519	coil	66.40982
3hvm	P 32 2 1	710.4	160	40	68.88373	22	0.74	30.45	6	76	4	7.021748	coil	67.65907
1zcm	P 21 21 21	709.04	151	45	48.17006	15	-1.05	25.48	12	80	6	11.76185	alpha	50.9527
2gwm	P 32 2 1	708.445	153	50	56.09469	15	-2.47	25.63	10	58	3	11.39538	alpha	46.38257
1k77	C 1 2 1	708.01	164	40	47.56712	51	-0.98	45.49	6	149	4	4.495162	alpha	80.00593
1fw8	P 21 21 2	707.92	154	48	58.79266	23	-5.74	30.58	10	49	3	7.270412	coil	53.46508
1ys1	C 1 2 1	707.145	128	43	92.24841	25	4.51	29.925	5	40	0	6.667473	alpha	57.0774
3b0t	P 21 21 21	706.865	139	41	65.92277	26	1.08	31.745	8	67	1	6.703543	alpha	65.58324
1pa2	P 21 21 21	706.04	142	49	57.69432	20	-1.53	26.94	8	68	2	7.356694	coil	62.6735
3nyc	P 21 21 21	705.865	150	37	54.28517	23	-0.17	37.895	9	114	4	7.616711	beta	66.55239
1fcy	P 41 21 2	704.8	136	40	60.1582	14	-0.34	30.415	6	61	3	9.807037	alpha	45.06172
1eyh	P 31 2 1	702.18	154	38	50.00712	31	1.84	44.03	4	90	4	9.584081	alpha	78.17654
2v3i	I 2 2 2	697.48	156	60	50.51328	13	-4.48	26.1	12	74	4	9.416399	beta	49.26306
1rc9	I 2 2 2	695.06	150	36	62.39317	20	2.18	30.05	8	76	2	8.142995	alpha	64.56709
2abk	P 21 21 21	694.925	152	44	55.97151	22	-2.76	29.47	9	61	3	10.35435	alpha	61.23179
2frg	P 21 21 2	694.88	152	42	59.7319	19	1.12	32.21	7	76	3	4.584964	beta	70.64025
3psh	P 31 2 1	694.465	144	42	56.44345	26	-2.81	36.195	5	87	3	7.654266	alpha	74.97498
1fnf	P 1 21 1	692.6	140	42	44.03407	19	-1.14	26.76	11	98	5	10.47195	beta	59.31634
1i9e	I 41 2 2	691.4	134	44	59.12786	6	1.9	29.07	10	62	3	4.115013	beta	8.779288
1qi7	P 43 2 2	687.63	152	42	68.45833	20	-0.02	27.37	8	47	0	12.6287	alpha	60.7856
1sbx	P 1 21 1	687.49	137	42	55.82772	18	0.26	33.435	5	66	4	5.984262	alpha	56.48955
1ud2	P 2 3	685.93	172	55	52.24294	17	-5.24	32.88	12	57	1	8.305512	coil	43.39583
3fhf	C 1 2 1	685.77	168	36	50.62485	18	0.56	41.12	6	77	2	4.124196	alpha	56.48687
1qnr	P 1 21 1	683.135	152	49	47.42913	11	-2.86	27.895	10	55	2	13.36888	coil	49.48729
2rku	P 32 2 1	679.12	132	38	66.7452	12	0.48	29.24	8	55	1	11.86756	alpha	32.64666
1g43	P 65 2 2	676.07	158	48	46.16682	18	-0.42	32.43	8	57	2	5.765853	beta	56.57994
2azy	P 31 2 1	672.9	135	42	57.90756	19	2.49	31.715	8	48	3	7.65882	alpha	33.49532
3fwa	P 41 21 2	672.21	144	41	57.44633	17	-0.15	28.515	9	69	4	6.572158	alpha	56.57905
3gbl	P 21 21 21	671.6	154	42	58.21694	34	1.13	39.865	8	79	3	4.070503	beta	74.20116
2jg6	C 1 2 1	670.43	147	35	56.51298	18	-1.7	35.395	4	80	1	6.51913	coil	57.08575
1xmk	P 21 21 21	667.4	147	42	59.8172	27	-1.13	30.83	5	59	3	4.086005	alpha/bet	66.13575
2ov3	C 2 2 21	667.08	140	40	65.86017	25	-3.15	37.49	6	53	0	4.184086	alpha	58.67212
2dyi	P 21 21 21	666.99	143	40	57.03309	27	0.55	34.905	8	40	1	6.922143	beta	72.64352
1sdi	P 21 21 21	666.665	144	37	59.00865	26	1.62	37.485	5	74	3	9.355523	alpha	73.44093
3emc	P 21 21 21	665.035	138	38	48.51624	34	-1.08	35.375	6	72	4	9.231274	alpha	67.83177
1zzm	P 1 21 1	663.395	145	43	50.46164	14	-1.33	27.715	9	55	2	9.732316	alpha	44.31598
1a8s	P 2 3	662.96	158	40	66.47234	32	0.15	39.35	7	67	2	6.490769	alpha	82.90395
1wd5	P 41 21 2	661.915	143	48	52.1434	15	-3.97	27.655	11	60	4	11.64065	alpha	48.13609
3le2	P 43 2 2	661.42	138	46	58.34719	14	-0.82	26.35	8	81	0	17.72152	alpha	42.46923
1z6i	P 41 3 2	661.385	129	36	56.43234	23	0.05	31.89	7	67	0	6.49602	beta	66.15133
3mwz	I 2 3	659.815	135	38	51.67585	30	-1.93	35.33	7	103	8	4.670051	beta	70.26742
1bmg	P 21 21 21	658.14	153	38	59.77224	24	1.88	36.695	7	60	3	4.917829	beta	68.01061
2a6z	C 1 2 1	657.15	139	47	53.6917	27	-3.46	28.87	13	68	1	6.917934	coil	73.32877
3nd2	P 1 21 1	656.835	151	46	44.23485	13	-2.9	22.385	12	65	5	10.51291	alpha	49.8291
2bmm	P 64 2 2	656.6	132	34	61.02193	30	1.84	38	6	98	6	5.448523	coil	65.2254
1p4x	P 65 2 2	656.35	160	40	60.30662	17	-4.3	20.3	8	50	2	15.81702	alpha	65.37975
3qmx	P 21 21 21	655.96	141	39	54.57703	31	0.2	36.575	6	83	2	5.067946	coil	64.93689
1bsg	P 21 21 21	655.675	152	44	38.81954	18	-2.68	33.14	8	80	8	5.759896	alpha	56.53334
2qjl	P 21 21 2	653.82	130	38	59.21049	20	0.06	31.45	8	98	4	4.191521	beta	54.91114
1e29	C 2 2 21	652.59	134	36	50.58	16	-1.22	33.07	6	52	1	8.466066	coil	59.57339
1hlb	P 1 21 1	651.115	140	38	58.78531	19	-1.4	28.38	7	30	0	9.236848	alpha	53.37997
3od3	P 1 21 1	650.745	125	47	67.30901	12	-0.68	24.27	11	45	1	17.43655	beta	20.94061
1h6h	C 1 2 1	650.3	124	46	73.93511	15	1.42	27.1	4	30	4	10.20337	alpha	29.1896
1xt0	P 21 21 21	649.91	139	45	56.34011	17	-4.28	25.08	10	64	1	12.2867	alpha	62.62098
2rj2	P 32 2 1	648.53	140	34	69.53264	44	2.78	43.83	4	94	2	3.262949	alpha	79.35639
2ehg	P 43	647.87	134	37	51.50262	15	-0.4	30.8	7	81	4	8.053699	alpha/bet	52.76213
2pnd	P 21 21 21	646.685	140	44	54.78865	20	-3.23	32.325	7	52	2	5.594687	beta	57.19864
1fbn	C 1 2 1	646.15	136	32	60.56488	24	-1.7	34.09	8	81	12	4.570332	beta	82.46073
1cdy	C 1 2 1	645	132	40	47.35659	17	-3.74	29.73	8	109	2	9.575581	beta	51.75504
3lp5	P 21 21 2	643.53	133	39	47.76079	10	-3.7	31.21	10	53	1	8.14997	alpha	32.06533
1tib	P 21 21 21	643.285	130	40	86.43836	17	1.56	28.495	8	34	0	7.056546	alpha	58.59922
3lrp	C 2 2 21	640.46	134	30	63.2561	34	2.08	37.91	6	63	0	4.405427	alpha	72.98036
3mx7	P 31 2 1	640.0025	138	34	71.09113	25	3.81	39.14	5	56	1	3.148222	coil	79.91766
3oos	I 2 2 2	639.9	144	34	61.20175	24	1.58	29.42	6	83	4	5.395562	beta	65.99781
1tca	P 21 21 21	637.145	115	39	88.13143	15	1.89	18.72	7	22	1	9.259274	alpha	45.11767
3bod	P 21 21 21	636.555	128	39	58.2432	25	0	34.225	5	55	1	7.544705	beta	57.72007
1f00	P 1 21 1	636.005	142	43	64.94053	27	-2.15	34.55	8	53	3	6.129071	beta	69.87209
1tqg	P 2 2 21	635.64	142	34	55.31433	30	1.12	36.48	4	74	2	6.891873	alpha	74.97955
2pet	P 1 21 1	635.595	148	47	51.97492	9	-3.52	17.65	13	43	2	14.79362	beta	30.23938
1r6j	P 1 21 1	635.33	128	42	69.09244	27	-0.11	33.675	7	67	4	3.893835	beta	61.69314
2rkm	P 21 21 21	634.785	136	53	41.32817	10	-7.03	21.775	15	50	3	22.42077	coil	38.77533
3hbn	P 41	633.27	143	39	56.54381	32	-0.8	35.07	6	65	1	7.002937	alpha	72.5441

1b6g	P 21 21 2	633.14	133	42	45.79951	20	-2.75	31.01	6	83	3	8.374325	coil	56.75127
2hwk	P 21 21 21	631.21	126	39	45.71062	12	-1.79	26.715	9	65	1	13.4153	alpha	51.76645
1rw1	C 1 2 1	630.655	142	38	60.75588	35	-1.98	40.795	6	70	2	5.282	alpha	70.38872
2ux7	H 3 2	630.29	124	46	60.86563	19	-4.04	33.13	4	44	0	4.696045	coil	45.31089
1qfm	P 21 21 21	629.07	130	37	40.93503	12	-0.91	28.465	9	75	1	13.84722	alpha	44.99897
1k12	H 3 2	628.645	135	44	46.22402	19	-2.42	31.395	5	51	4	8.12282	alpha	51.887
1flj	P 1 21 1	628.075	135	42	56.48848	16	-3.31	28.96	10	55	2	9.914214	coil	36.8491
1ij5	C 1 2 1	627.7	144	36	60.75832	32	0.3	37.54	7	68	2	10.48471	alpha	77.16186
1elt	P 41 21 2	627.69	138	46	45.04532	14	-2.6	29.66	12	75	8	7.570616	beta	32.91832
3h8n	P 21 21 21	627.435	124	41	53.3099	24	0.39	31.375	11	65	2	8.224549	beta	65.94548
1l8f	P 41 21 2	626.98	138	48	56.44837	32	3.8	34.9	12	66	2	6.368018	beta	58.03694
1ng6	P 1 21 1	626.44	125	42	50.79018	17	-4.44	24.05	7	77	4	6.879941	alpha	53.1695
1iv8	P 1 21 1	626.24	150	48	49.51616	14	-1.7	25.515	12	78	1	19.73025	alpha	40.20104
3o7t	I 2 2 2	625.82	118	34	54.96309	22	-2.96	34.41	8	81	8	6.557796	coil	73.92062
1mc2	C 1 2 1	625.72	140	48	48.55047	10	-4.36	23.06	8	55	6	10.04862	alpha	40.65237
2e7z	C 1 2 1	625.53	158	56	42.12907	4	-5.76	17.75	16	48	4	20.9882	alpha	13.54371
1bxo	C 1 2 1	625.02	122	38	59.55329	5	2	21.41	8	48	4	10.21103	coil	22.35448
2apr	P 21 21 21	624.92	132	42	47.91253	20	-2.91	34.695	8	72	2	8.570889	coil	62.74723
2uwf	P 21 21 21	624.875	149	43	58.09242	16	-5.06	25.1	11	53	2	14.96099	alpha	43.5135
3ic4	P 21 21 21	623.945	124	38	54.54567	35	-1.54	33.855	6	85	7	4.554488	alpha	74.99379
3a4c	P 41 21 2	623.87	123	31	52.17433	20	0.05	33.95	5	53	3	5.701909	alpha	50.86797
1izd	P 21 21 21	623.405	139	48	49.10532	17	-4.37	32.75	9	61	1	7.95831	beta	60.9724
3a09	P 1 21 1	622.69	141	45	55.12615	16	-4.14	25.885	6	48	3	17.4796	alpha	38.48384
1fob	I 2 2 2	622.51	154	46	53.92524	13	-0.14	34.23	10	63	0	13.33714	coil	49.88354
1k7j	P 31 2 1	620.535	135	37	59.91604	16	-3.52	30.43	10	70	4	10.6384	alpha	56.53992
2qcp	P 21 21 21	618.195	127	34	57.33142	24	-1.66	36.405	6	61	5	3.199233	beta	74.27349
3i06	P 65 2 2	617.675	137	41	63.00644	32	1.34	36.14	8	71	1	7.152418	coil	52.58429
1pmi	C 1 2 1	617.28	130	43	49.2224	14	-6.93	31.13	11	43	0	7.506917	coil	54.78875
2i6j	P 41	617.16	133	38	58.27257	27	-1.93	32.845	7	64	2	5.359226	alpha	69.48765
1kvs	P 32 2 1	615.61	138	44	77.97632	12	2.12	29.33	4	38	0	8.618281	alpha	41.78295
2qdx	P 21 21 2	615.46	154	40	58.31248	16	-3.32	33.92	8	54	4	11.71867	coil	59.96328
1x46	C 2 2 21	613.82	118	38	70.71617	27	-1.32	24.92	4	34	0	9.908035	alpha	77.15943
1cgt	P 21 21 21	612.38	126	44	48.69607	15	-0.72	32.37	9	52	5	9.137709	coil	48.04125
2oj4	C 1 2 1	612.01	118	36	57.7703	19	-1.52	23.05	8	47	0	10.73144	alpha	48.47633
2plc	P 31 2 1	611.44	109	37	71.43056	22	1.17	27.795	8	20	1	7.733171	alpha/bet	65.15684
1ad6	P 21 21 21	611.035	113	31	68.53126	31	3.4	30.315	7	68	1	7.114159	alpha	72.20208
1pml	P 21 21 2	610.995	129	41	53.13955	22	-3.15	32.74	8	78	4	8.037087	beta	54.09373
2xyl	P 1 21 1	610.925	143	42	50.0888	20	-4.39	24.65	11	56	3	11.12616	beta	59.66281
1zt3	P 21 21 2	607.57	136	48	53.10664	37	-1.86	35.12	6	78	2	8.282371	coil	79.57272
3bjo	F 2 2 2	607.56	122	44	81.93759	23	2.66	30.56	8	20	0	7.915877	alpha	64.61913
1hp1	P 41 21 2	606.985	126	41	70.84524	20	-0.49	33.575	9	32	1	14.37329	coil	61.17696
2b8i	F 2 2 2	606.9	112	38	72.20959	20	-0.14	28.75	6	51	0	3.086373	alpha	50.74642
2xt0	P 21 21 2	606.86	138	42	72.37584	30	1.8	32.41	8	39	2	8.68136	alpha	86.92779
3p3g	P 61	606.835	134	37	46.56785	20	0.03	25.545	11	76	2	12.93638	alpha	59.50382
1bd8	P 1 21 1	603.22	133	36	58.54995	24	-1.54	28.01	8	75	2	7.312622	coil	68.72037
1gk7	P 62 2 2	603.07	126	30	72.03973	24	0.28	27.27	2	73	1	3.078001	alpha	74.45239
2hvm	P 21 21 21	602.93	138	47	54.26418	9	-1.79	26.43	13	37	1	12.43241	alpha	40.97076
2wbx	C 1 2 1	601.34	132	32	54.84252	9	0.94	27.58	8	69	8	5.696644	beta	40.4613
1agi	P 21 21 21	601.1	123	32	44.74048	20	-2.31	29.55	6	52	3	8.399601	beta	64.81035
1ccz	P 32 2 1	599.95	126	32	53.62864	37	-2.96	38.48	7	62	0	7.166847	beta	65.92299
2ahn	P 1 21 1	599.845	138	45	54.65995	14	-0.88	31.29	10	64	4	9.964441	beta	56.95221
3iv4	C 1 2 1	598.58	117	36	55.84968	20	0.94	30.565	5	49	4	5.14217	alpha	59.14999
2ja4	P 32 2 1	597.84	135	37	53.00666	27	0.13	32.59	9	93	4	6.401796	coil	61.79245
2xw9	P 1 21 1	597.41	129	40	54.12447	14	-0.92	29.39	10	54	2	11.10104	coil	52.6481
1b9o	P 21 21 21	595.595	130	39	57.00182	25	-1.88	36.795	6	54	3	5.258607	alpha	57.68433
1gq8	P 21 21 2	594.54	133	41	50.20604	15	-0.22	26.43	9	55	1	7.742961	coil	54.0771
1axn	P 1 21 1	594.095	136	46	59.82882	11	-3.82	18.565	10	53	3	14.59994	alpha	26.27105
1nwz	P 63	593.7	130	37	57.43305	37	-0.46	36.5	7	64	1	4.576166	coil	91.15715
1am5	P 21 21 21	590.23	126	45	62.05971	18	-0.86	25.475	13	50	0	11.81932	alpha	40.12927
1ogq	P 21 21 2	589.88	134	42	54.27206	18	-1.52	22.12	14	56	2	13.81742	coil	57.21164
1ce2	P 21 21 21	588.66	123	37	43.52937	13	-4.4	25.37	8	53	2	20.32475	coil	38.79829
3m5q	C 1 2 1	588.02	125	39	57.41386	20	-1.49	31.33	9	46	3	10.68139	coil	55.45662
1itx	P 1	587.505	130	38	63.3935	28	-0.41	32.965	8	65	2	12.78755	coil	54.59613
3a3c	P 21 21 21	587.15	132	43	62.07869	24	-4.71	26.335	11	53	1	16.04295	alpha	59.49587
1hfx	P 21 21 21	586.995	126	38	54.66997	32	-3.28	37.035	7	77	3	5.789862	alpha	66.96991
2qnk	P 21 21 21	586.36	130	38	43.69841	14	-2	33.865	7	75	9	7.275394	alpha	50.13302
3nju	P 41	584.335	137	38	51.87264	18	-1.44	31.905	6	49	4	8.721453	alpha	63.5329
2sfa	P 21 21 21	584.305	122	44	47.45638	19	0.82	31.495	9	56	3	9.160883	beta	58.01251
2icc	P 21 21 21	583.975	132	43	46.27767	12	-3.73	28.595	9	49	3	9.680414	beta	39.88013
1ew4	P 32 2 1	582.82	134	34	54.76476	25	-4.06	37.13	4	85	1	7.927611	alpha	62.8942
1bk9	P 61	582.195	125	36	51.01212	22	-1.6	37.195	5	73	2	7.89555	coil	57.23254
3lez	P 31 2 1	580.76	134	44	55.47214	15	-2.62	23.25	8	61	2	15.71389	alpha	42.80081
1g12	C 1 2 1	580.705	125	40	59.29517	28	1.19	33.28	7	64	2	6.794104	coil	62.63249
1t4w	P 21 21 21	579.3	136	35	58.07613	29	-3.3	37.98	6	71	4	10.1314	beta	72.88624
1qm7	P 31 2 1	579.05	114	38	59.9102	19	2.32	31.16	6	29	0	2.133235	beta	46.76107
1bhe	C 1 2 1	578.6	126	46	58.18009	8	-4.54	23.14	12	30	2	16.10503	coil	44.07363
3e4g	C 1 2 1	577.905	118	40	42.10813	12	-1.91	27.645	12	65	1	10.8917	beta	40.17702
1r8n	P 21 21 21	577.05	129	44	48.77134	13	-4.09	25.935	8	46	2	10.902	coil	32.29616
3l6g	P 41 21 2	574.915	128	48	57.06844	9	-4.89	21.14	9	38	2	16.70725	beta	38.26653
3ld1	P 64	574.575	121	37	71.25267	26	0.96	23.38	8	39	1	8.97533	coil	68.47757
2qsv	P 31 2 1	574.28	126	38	69.14745	16	-3.1	21.59	8	37	0	10.10831	beta	62.58619
2v8i	P 21 21 21	573.89	125	35	49.1819	11	-2.33	26.175	8	44	1	20.38913	alpha	46.53592
2qht	P 41 21 2	573.78	121	39	56.54432	30	0.94	32.14	5	59	3	7.87605	alpha	57.6972
1kkh	P 1 21 1	573.25	128	36	48.55997	13	-4.84	21.195	10	57	3	16.86175	coil	44.61579
1g1t	P 21 21 21	572.305	125	35	57.37151	18	0.05	29.225	8	49	1	10.44985	alpha	47.20472
2y44	P 1	570.355	124	30	51.69237	12	-4.8	25.96	6	62	5	10.36723	alpha	57.22752
1ks5	I 4 2 2	570.22	122	46	53.79152	13	-3.08	30.23	10	45	0	9.168163	beta	39.95475
2apb	I 21 21 21	570.15	126	36	55.06621	32	1.48	30.67	8	59	8	6.061563	beta	74.90134
3akb	P 21 21 21	568.5	119	33	43.02639	18	-4	30	6	70	2	4.125981	coil	46.44151
2dri	P 21 21 21	567.925	117	42	53.7254	19	-0.78	22.3	9	37	1	14.64278	alpha	59.26575
2dwr	P 32 2 1	567.64	132	42	42.48291	21	-3.03	28.63	8	66	4	11.42758	beta	60.13406
3deo	P 21 21 21	567.28	128	39	67.40146	20	-1.91	28.02	10	57	0	10.47102	beta	43.19913
1j09	P 21 21 21	566.515	127	47	46.07822	17	-3.88	22.985	8	59	5	17.75923	beta	45.09766
1m61	P 1 21 1	566.495	114	35	54.06932	15	-1.81	27.105	6	55	2	12.20729	alpha	56.82751
3caf	P 21 21 2	566.21	124	34	46.24433	21	0.32	30.45	6	62	4	5.173875	beta	64.74983
2nwd	P 21 21 21	565.985	115	34	46.39964	22	-0.68	35.735	6	71	2	6.302941	beta	51.45808
1y0m	P 1 21 1	565.735	118	32	59.23975	29	0.04	33.785	7	56	3	5.392984	beta	64.26242
1x38	P 43 21 2	564.815	116	39	57.49759	16	-2.09	21.83	9	45	2	13.43272	alpha	52.45169

3f7m	P 21 21 21	564.38	124	40	43.17747	23	-0.24	29.985	7	71	1	9.651742	alpha	63.79124
2xu9	C 2 2 21	563.185	126	41	44.33623	9	-2.07	21.62	10	68	6	16.31812	coil	26.6076
2ywz	C 1 2 1	563.07	122	40	40.87946	13	-3.26	25.05	6	76	6	6.467437	beta	39.39297
1cyg	P 21 21 21	562.4	135	42	50.81881	21	0.35	28.8	8	53	3	15.96883	beta	42.50356
1bg4	P 31 2 1	562.23	120	39	59.16796	25	-1.66	29.09	6	75	3	9.010316	alpha	65.84227
1f20	P 21 21 21	560.57	132	40	41.02342	8	-5.65	21.51	13	53	2	16.91202	alpha	36.01245
1zin	P 1 21 1	560.46	127	35	57.73204	20	1.61	32.505	8	34	0	8.695536	alpha	75.56918
1wza	P 21 21 21	560.435	127	44	58.48225	13	-2.48	27.66	9	44	1	20.58958	alpha	40.44448
3jyz	P 41 21 2	558.66	126	36	63.66484	19	-0.54	33.81	8	45	0	9.720349	alpha	57.4804
2v9v	C 1 2 1	558.62	119	32	70.89524	28	1.4	28.2	7	52	0	5.95104	coil	80.07948
3mp2	P 41 21 2	557.87	96	28	61.74378	31	2.18	23.96	4	28	2	8.318461	beta	80.80377
1jbe	P 21 21 21	557.37	110	29	52.94598	11	-2.16	27.995	6	53	2	6.291368	alpha	38.9149
2fyg	I 21 3	557.31	120	34	52.80544	23	3.56	23.5	8	101	2	6.510057	coil	68.88985
2rbk	P 21 21 21	557.255	121	38	53.90979	20	-3.1	27.555	9	42	1	9.448098	alpha	59.70426
2p3k	P 41 21 2	557.13	114	36	52.09556	33	-2.98	28.39	8	56	3	8.274999	beta	70.92779
1jug	P 1 21 1	555.63	124	33	60.31982	26	-1.23	35.965	6	76	2	6.821302	alpha	66.3931
1dqy	C 1 2 1	555.46	130	38	48.71278	28	-0.18	30.18	6	73	1	8.80418	alpha/Bet	61.0539
1mdc	P 1 21 1	554.55	118	36	50.15959	23	-2.97	31.48	6	55	3	7.236264	beta	50.79344
1qcx	P 21 21 2	554.31	131	35	57.03307	33	0	35.935	7	41	2	11.59887	alpha	63.24981
3mu7	P 1 21 1	554.13	131	44	56.52554	15	0.51	33.59	10	55	3	10.12871	alpha	51.28039
1i27	P 21 21 21	554	115	32	64.62816	19	-1.2	26.575	6	46	1	6.347924	alpha	61.17238
3mao	P 21 21 21	553.42	125	36	64.81425	24	-0.13	37.725	10	74	5	6.744877	coil	59.32384
1vbl	P 32 2 1	552.55	116	30	65.63207	17	-3.86	34.32	6	72	0	9.956113	alpha	70.33753
2g5x	P 1 21 1	552.355	124	40	53.84218	14	-0.48	22.94	8	47	2	13.79071	alpha	43.18871
1gqv	P 21 21 21	550.595	117	31	50.56167	17	0.32	32.43	7	47	2	8.385474	alpha	64.19237
3mi4	P 21 21 21	550.43	121	37	52.64611	17	-0.4	29.115	10	75	6	6.474938	beta	59.78962
2zk9	P 65 2 2	550.39	120	34	45.902	13	-1.02	27.77	10	35	0	7.763132	alpha	38.87789
2hxs	P 21 21 21	550.33	114	37	61.01975	11	-2.42	24.535	6	43	1	14.50993	coil	47.85856
1wp5	P 32	548.78	128	36	49.63556	20	-2.5	33.54	10	56	3	15.67021	coil	67.09428
3mgw	H 3	548.575	125	39	56.77619	22	-2.99	27.41	7	53	2	9.296122	coil	56.27489
1esw	P 64	548.55	112	32	54.23115	19	-2.12	33.76	6	61	2	8.016808	beta	55.5674
1y6i	P 21 21 21	548.255	114	38	59.19052	12	0.72	26.745	5	53	3	8.045745	coil	29.99881
3a3j	P 21 21 21	548.17	119	30	59.37665	36	-2.93	37.34	4	56	2	14.9242	alpha	77.56718
3n6m	P 32 2 1	548.095	126	40	62.04125	11	-0.39	25.04	9	32	1	13.49766	alpha/bet	37.36396
1npc	P 65 2 2	547.85	123	48	53.8213	11	-1.88	22.225	8	36	2	17.19379	alpha	42.90408
2ggc	P 1 21 1	547.775	117	35	59.11825	24	-0.96	32.6	6	45	0	10.30167	alpha	67.04212
1dxj	P 61	546.78	116	44	55.451	14	-1.65	26.795	7	37	3	9.974761	coil	42.77955
3bmv	P 21 21 21	545.1	130	39	46.46762	18	-2.49	30.33	11	44	2	16.71092	alpha	34.60191
1ae7	P 31 2 1	544.75	130	44	70.06333	5	-1.06	20.03	14	11	1	11.99449	alpha	29.61909
1is1	H 3 2	543.06	105	32	38.27386	9	-1.52	29.66	5	55	3	7.631753	alpha	30.09704
1ah4	P 43 21 2	542.39	121	36	55.80118	16	-4.11	28.505	7	59	2	10.97019	coil	45.66732
2acy	C 1 2 1	541.2	126	30	54.46231	44	-2.5	38.63	6	68	2	2.718958	beta	83.84331
3po0	P 21 21 21	539.595	131	36	60.27947	17	-3.19	32.08	5	55	3	4.659736	alpha	57.25776
1xv5	P 1 21 1	539.53	113	40	62.97611	5	-2.98	21.74	6	28	0	12.64224	coil	29.34221
1llp	P 21 21 21	539.36	114	37	64.22148	15	-2.37	27.35	7	34	0	10.26216	coil	45.87381
2go2	P 21 21 21	538.79	111	35	55.77127	27	1.72	31.595	10	63	3	10.59875	beta	73.96852
1zwp	P 43	538.765	122	31	64.89471	20	1.16	37.06	5	48	3	5.512608	alpha	62.13841
1yqe`	P 21 21 21	537.97	129	31	50.53813	7	-1.13	31.84	9	50	2	15.56475	coil	41.0692
1bz4	P 21 21 21	536.605	111	30	54.19163	25	-0.61	32.93	4	71	7	6.641757	alpha	69.78411
2bk8	I 41	535.78	96	32	60.52298	10	0.16	23.75	6	49	2	10.15435	beta	15.69114
2oq5	P 2 21 21	535.715	121	36	62.62845	11	-1.21	26.755	9	44	1	12.80158	coil	52.6306
2w15	P 21 21 21	532.845	113	36	50.05771	10	0.19	26.095	9	41	3	9.608554	coil	18.48474
2pmk	C 1 2 1	532.76	100	30	45.92875	16	-0.56	30.48	4	45	0	6.41728	alpha	63.21608
2eek	P 21 21 21	531.6	107	34	53.10384	8	-1.34	28.3	7	57	3	5.129797	coil	28.04364
1mwp	P 21 21 21	530.145	103	31	66.26678	29	1.27	30.99	6	66	2	7.792208	coil	54.30778
2wll	P 65 2 2	530.12	100	32	53.48412	16	2.06	29.32	7	69	4	8.397382	beta	50.43386
2of3	P 41	529.755	103	35	41.31061	16	-3.39	26.185	6	63	6	11.36563	alpha	51.04907
1hx0	P 21 21 21	529.295	117	44	52.33376	9	-3.07	20.86	12	48	2	17.16529	coil	19.97468
1qtw	P 1 21 1	528.02	116	31	53.20726	29	-3.08	34.81	6	47	1	10.04791	alpha	72.6516
2abl	C 1 2 1	528.015	126	36	62.64689	31	1.97	35.1	5	54	1	7.120536	alpha	72.47805
2qen	C 1 2 1	527.86	128	29	47.30232	13	-0.32	25.22	9	58	1	11.75808	alpha	56.67885
2gsj	P 21 21 21	526.365	129	41	45.9396	17	-1.1	28.225	9	57	1	12.38137	coil	59.53568
3h0o	P 21 21 21	525.2	106	34	41.2024	28	-3.18	31.6	7	79	7	9.677742	coil	68.0436
1yw5	P 1 21 1	524.96	126	38	40.86692	17	-4.13	24.755	11	47	0	13.89963	alpha	57.83203
2erf	P 1 21 1	524.725	102	33	43.13879	14	-2.46	27.275	7	74	3	11.481	beta	42.70046
3c7f	P 21 21 21	524.66	120	38	50.42027	16	-1.74	28.015	8	53	2	16.38417	coil	51.3199
1cjw	C 2 2 21	523.54	120	32	59.81014	20	1.6	29.8	8	33	0	11.71328	alpha	73.84727
1g8a	P 1 21 1	523.235	114	26	41.66961	11	-1.69	27.085	7	75	5	14.79689	coil	36.41863
1zua	P 61	520.885	119	34	56.24274	8	-2.87	23.625	9	44	0	19.92401	coil	48.44735
1h2e	P 41 21 2	520.37	102	34	42.46209	12	0.94	23.96	6	71	4	11.33063	alpha	36.47597
2evb	P 21 21 21	519.795	102	31	61.23857	32	-0.04	31.785	6	49	1	6.525399	beta	62.41403
2bnh	I 4	518.995	113	33	73.33693	26	-1.31	32.935	6	15	0	11.28261	alpha	58.2626
1gyc	P 21 21 21	518.605	94	38	67.12238	12	-0.02	20.29	14	25	1	17.21975	coil	38.08679
2cy2	P 31 2 1	517.92	122	38	64.6316	23	-2.86	29.41	6	39	0	6.842273	coil	56.3253
1qst	P 32 2 1	517.21	110	34	68.92751	35	-2.22	32.76	4	45	0	6.525396	alpha	55.92313
1nio	C 1 2 1	516.22	104	34	62.69807	18	-1.21	25.875	7	30	2	7.838945	alpha	46.93542
1jhs	P 21 21 21	516.005	104	35	61.74649	24	0.5	25.395	7	47	3	12.2506	coil	60.78914
1vb0	P 41 21 2	515.01	108	32	47.99518	30	2.24	32.07	4	62	8	3.197996	beta	55.18534
3d30	I 2 3	514.665	121	33	65.79037	27	-1.76	34.69	5	46	1	4.701835	beta	66.00507
1jfx	C 1 2 1	514.06	114	40	45.97518	19	-0.82	23.96	6	32	0	9.014259	alpha	43.30039
1kao	P 1 21 1	513.315	110	33	48.47219	12	-2.33	34.135	8	56	1	8.849829	coil	58.63261
1k40	C 1 2 1	512.265	106	29	68.93893	31	0.82	32.435	4	28	0	6.324851	alpha	61.87715
2gv0	P 21 21 21	511.825	100	32	48.81063	19	-2.13	29.21	5	69	4	5.782973	alpha	54.93382
1g9g	P 21 21 21	511.55	98	32	65.69739	9	-3.75	21.02	6	25	1	14.99632	alpha	34.4414
3kje	P 32 2 1	511.5	128	40	61.97165	11	-0.06	25.16	4	34	0	22.4538	alpha	48.61584
1oc7	P 21 21 21	510.755	112	35	63.25342	14	0.34	20.49	10	37	0	18.41611	alpha	54.70137
1i71	P 21 21 21	509.595	109	32	59.48449	28	0.97	32.945	6	65	4	3.139258	beta	77.78628
1lsl	C 1 2 1	509.07	112	32	67.28446	34	1.82	35.43	5	53	3	6.119237	beta	82.69491
1lsg	P 21 21 21	507.945	116	36	50.67675	21	-0.66	29.055	8	26	0	7.175974	beta	66.58989
2iim	P 32 2 1	507.8	102	28	56.51635	29	1.5	31.14	6	70	0	2.937672	beta	69.70658
1lba	C 2 2 21	507.63	107	30	58.4402	14	-2.39	22.94	7	26	3	8.928984	coil	39.41946
2hd9	P 32 2 1	507.2	116	30	51.23028	28	-0.52	29.83	8	22	1	4.711159	beta	59.44992
1g3q	P 21 3	506.905	108	32	54.74103	38	-1.58	34.5	7	65	3	6.145372	alpha	73.82942
1dvo	P 21 21 21	506.61	116	32	49.8184	17	-1.73	25.9	6	41	2	9.306705	alpha	63.8124
1z16	P 64	506.37	122	34	54.7406	5	-5.28	25.34	6	32	0	19.84195	alpha	31.2558
1w8o	P 21 21 21	506.25	109	35	67.9279	16	-2.44	30.5	9	39	3	7.546667	coil	41.2484
1thg	P 1 21 1	504.38	118	38	54.38063	14	-2.37	24.96	10	53	3	14.65413	alpha	41.57183
3agn	P 1 21 1	504.195	116	31	43.63421	28	-0.18	36.12	6	74	4	8.969744	coil	63.95839

3fci	P 21 21 21	503.355	111	35	71.24097	23	1.76	27.865	7	36	0	11.09678	coil	52.99143
2p71	I 41	501.385	119	33	58.4102	17	-2.07	31.175	5	55	3	9.006552	alpha	55.43844
3ip0	C 1 2 1	501.02	114	34	53.34118	9	-1.9	21.09	12	59	2	14.27412	coil	23.29847
2in0	P 21 21 21	500.755	110	39	41.99758	7	-1.82	22.91	10	44	3	11.67337	beta	24.70869
1tjy	P 21 21 21	499.325	101	30	52.77725	37	-2.98	35.395	3	78	4	6.907826	alpha	77.50563
3mf6	P 31 2 1	499.14	111	36	51.19706	21	-0.79	29.715	10	41	3	5.368734	beta	40.74809
2pi6	P 21 21 21	498.76	117	33	50.7248	14	-1.29	24.595	10	40	1	15.48915	alpha	61.67395
2ns0	C 2 2 21	497.965	114	29	56.01799	20	-2.2	33.97	7	63	0	6.784352	alpha	73.25013
1xk5	P 41 21 2	497.875	115	28	58.37309	24	1.2	31.795	5	48	0	12.3442	alpha	54.39016
1o73	P 1 21 1	497.72	109	29	65.61621	15	-1.47	30.375	6	32	0	8.883007	alpha	61.41807
1j8r	I 2 2 2	497.715	106	29	63.12147	25	-1.14	31.715	11	35	1	10.34779	beta	66.68676
1dsl	P 21 21 21	497.22	120	30	50.26648	18	1.94	32.88	7	54	2	6.733438	beta	70.49495
1zvg	P 21 21 2	496.48	114	34	49.96979	33	3.14	32.88	6	83	6	6.322007	coil	67.85168
1g6s	P 21 21 21	495.675	121	39	56.42306	17	-2.78	22.32	10	37	1	18.67679	alpha	46.53654
3a7l	C 1 2 1	495.31	100	30	53.27775	6	-0.36	23.04	10	49	10	7.215925	beta	38.37193
1aun	C 1 2 1	495.03	126	35	51.59586	26	0.52	34.925	5	72	3	10.47209	beta	77.74478
1oa4	P 21 21 21	493.92	113	35	55.03827	25	-0.66	33.72	8	49	3	9.19734	coil	68.52223
2fy6	C 1 2 1	492.34	104	30	75.44177	17	3	31.77	8	34	0	7.581651	coil	81.70776
2pth	P 21 21 21	492.05	97	33	66.81943	39	1.8	33.44	7	49	4	12.42861	coil	79.64943
1qj8	H 3 2	492.03	116	30	84.81393	22	5.28	29.48	8	33	2	12.70349	beta	74.9121
3p2j	P 21 21 21	491.885	110	34	55.4652	18	-2.81	29.545	7	46	1	9.667646	alpha	57.81229
1m40	P 21 21 21	490.59	107	39	49.13879	7	-4.19	21.26	8	48	1	13.76581	alpha	20.67612
1ouo	P 1 21 1	490.22	112	34	57.4589	21	-1.9	32.455	7	38	5	13.9139	alpha	48.78932
1edg	P 21 21 21	489.935	102	34	47.05012	15	-0.85	21.21	7	36	5	12.04827	coil	43.53026
3px8	P 1 21 1	488.97	108	34	53.26094	24	-1.7	27.085	9	46	2	14.80535	alpha/bet	69.6781
2x5o	P 41	488.69	109	35	51.92249	31	-1.26	31.795	5	61	5	10.48364	alpha	84.52598
1ly2	P 1	487.58	103	27	63.2809	26	1.91	36.255	6	44	2	10.14063	beta	76.4131
1iu9	P 21 21 21	487.185	114	28	74.2685	31	-2.21	36.28	4	35	0	4.89777	alpha	66.70361
2zeq	P 61	486.645	110	26	56.20113	39	-1.05	36.4	5	64	3	4.924021	beta	86.99052
1p6p	P 43 2 2	485.92	96	28	51.26358	10	-1.36	20.08	8	70	8	11.07127	beta	59.68061
1iqq	P 1 21 1	485.805	92	28	65.76301	14	-0.55	30.11	6	34	1	10.33052	coil	49.15655
3ga3	P 1 21 1	485.74	100	32	48.39523	18	-1.47	27.04	5	55	3	6.781406	beta	34.67905
2z4u	P 21 21 21	485.46	107	34	53.09191	20	-2.93	21.945	9	47	2	15.36429	alpha	56.98307
2i0w	P 43	484.395	102	34	53.18284	20	-0.43	27.875	8	34	3	12.55535	beta	56.43948
1prz	P 21 21 21	482.98	104	30	59.03764	12	0.08	24.435	8	30	1	11.02685	alpha	32.32536
1lqy	P 21 21 21	482.915	113	33	62.95104	16	-1.18	26.76	8	43	1	13.54431	coil	50.2956
2cyg	P 21 21 21	482.115	117	36	45.84591	32	-1.71	32.85	5	69	4	10.59861	alpha	72.70257
2fx5	P 43 21 2	481.805	110	38	62.12161	18	-1.71	24.085	7	42	0	6.801756	coil	57.67167
2bz7	P 61	481.375	109	32	61.54661	19	-2.36	31.8	7	62	1	6.969099	coil	58.8751
2os3	P 31 2 1	481.13	108	36	50.57261	8	-2.84	24.03	5	53	4	8.572008	coil	27.3211
1cpn	P 1 21 1	480.955	106	34	57.52409	19	1.58	30.64	6	56	2	7.396222	coil	55.10599
2hrg	P 21 21 21	480.39	114	42	60.36762	19	-1.68	28.32	7	38	2	18.74415	coil	63.76486
2agc	P 21 21 21	480.365	95	20	65.73647	19	0.17	31.655	5	56	3	8.986125	coil	67.20515
1ps9	P 21 21 21	479.255	103	35	49.03861	16	-1.94	26.015	8	47	2	13.9083	beta	49.25666
1cpq	P 21 21 2	478.02	110	32	70.10585	29	-3.98	32.62	5	46	1	10.03985	alpha	63.88436
1jln	P 21 21 21	477.98	111	33	54.42801	20	-3.35	31.035	7	47	2	14.91276	alpha	54.67907
3olg	P 43 21 2	477.62	112	36	55.90428	35	-0.99	32.125	6	38	1	11.72296	coil	84.79544
2oyp	P 1 21 1	477.45	113	35	54.68217	24	-2.66	29.29	6	50	3	10.93543	coil	66.09802
3gmu	C 1 2 1	477.42	108	39	58.81823	15	-1.64	19.84	10	41	2	12.5479	beta	38.64312
1i1w	P 1 21 1	477.175	105	31	53.3997	26	-2.21	27.875	6	67	5	15.36229	alpha	71.6341
1f82	P 21 21 21	477.115	102	34	42.14498	11	-1.75	28.775	8	51	2	16.18478	alpha	39.06186
2o6r	P 21 21 21	475.985	112	39	48.58346	16	-4.04	30.74	10	43	2	10.81309	alpha	59.2193
1r9l	P 21 21 21	475.595	120	41	52.23877	17	-5.14	21.64	9	58	2	21.35298	alpha	56.6585
1l2p	P 21 21 2	475.035	87	25	63.49216	23	-0.19	24.9	2	47	3	7.388929	alpha	62.13016
2fc3	P 21 21 21	474.375	98	29	62.9112	19	-4.01	29.51	6	54	4	8.345444	alpha	77.46614
3kb5	C 1 2 1	473.6	101	30	52.99937	22	-3.3	27.255	8	44	2	14.053	coil	46.33446
1hpt	P 43	473.515	102	23	65.60299	32	-0.97	37.52	4	75	1	5.673527	coil	73.24583
1xr6	P 32 2 1	472.185	96	28	52.97288	19	-4.18	24.18	8	40	1	13.80921	coil	42.93338
1zu0	P 21 21 21	470.975	123	38	55.46154	11	-4.02	22.565	10	27	0	18.66023	beta	56.80025
1gbg	P 1	470.37	104	37	55.3054	15	0.47	26.66	9	37	1	13.67594	beta	35.73676
2d81	C 1 2 1	468.32	104	38	69.42475	8	2.55	18.8	10	33	0	12.27285	coil	23.8683
1ruw	I 2 2 2	468.14	84	30	41.34874	14	-0.48	19.52	6	54	2	4.923997	beta	50.31401
3ct5	P 21 21 21	468.005	112	37	53.01653	20	-1.25	27.58	8	35	3	8.451833	alpha	51.51868
1htp	P 21 21 2	467.86	107	28	58.33476	25	-3.56	30.095	7	44	1	6.932352	beta	54.67448
2sli	P 1	467.78	111	42	39.08141	15	-4.04	27.445	11	61	8	40.34589	coil	42.8492
1ug4	H 3 2	467.295	98	32	59.78236	24	0.79	26.955	5	55	2	5.171252	beta	67.18347
1kex	P 41 21 2	466.56	111	31	65.92828	19	-1.17	29.43	8	40	1	9.852431	beta	67.38683
2c0h	P 21 21 21	466.18	105	35	61.18667	17	-1.91	28.785	5	39	1	11.04052	alpha	53.14793
1yp5	P 41 21 2	466.01	102	26	60.46222	22	-1.12	30.9	8	51	4	3.425613	beta	52.08472
1m4l	P 1 21 1	465.78	113	40	44.46949	11	0.43	17.675	8	44	0	17.57858	coil	23.20946
1ru4	P 21 21 21	465.35	100	33	49.00398	18	0.12	24.51	8	43	3	19.25566	coil	54.36553
3foj	P 21 21 21	465.055	95	23	64.61816	26	-0.59	33.385	5	37	1	4.992958	alpha	79.81314
1gny	C 1 2 1	464.69	108	34	66.85855	24	1.11	33.145	7	32	2	10.08817	beta	61.43235
1php	P 1 21 1	464.41	110	34	48.28384	11	-5.17	21.905	7	38	0	11.94014	alpha	37.35923
3aks	P 1 21 1	464.41	114	34	57.33834	36	0.08	38.33	9	75	1	7.114662	beta	66.72337
3fbl	P 21 21 21	464.275	90	27	63.14038	14	-0.74	28.385	5	44	0	5.757364	alpha	45.4332
1rl0	P 1 21 1	464.24	104	37	41.07143	11	-3.51	19.44	11	26	4	15.80486	alpha	36.40682
1sn4	P 61	464.04	95	32	58.16309	38	-1.14	33.72	6	49	3	5.302064	beta	65.11723
3p5u	P 21 21 21	462.015	111	29	55.90619	20	-0.02	33.295	11	44	0	12.8137	coil	60.71556
2d3y	P 21 21 21	460.825	106	29	55.83356	14	0.22	27.155	5	36	0	8.832529	alpha	59.02241
3gbo	P 1 21 1	460.46	96	30	59.0225	22	-1.48	25.362	7	51	2	12.10854	coil	57.39261
3etp	P 41 21 2	459.79	98	30	55.82005	29	0.34	33.295	6	55	2	5.461189	beta	56.40184
1lpj	C 1 2 1	459.47	110	32	40.45748	36	-1.68	37.93	6	76	2	7.191155	beta	95.16182
1xeu	P 21 21 2	459.195	95	29	55.9784	23	0.49	26.57	7	40	0	12.1713	beta	55.85318
2xa3	P 21 21 21	458.83	100	33	57.63899	29	-1.78	31.805	5	54	1	6.105202	beta	69.27729
1jmm	P 63 2 2	458.28	98	30	48.26525	10	1.36	24.65	6	59	8	18.54379	beta	42.60278
1rgy	P 21 21 21	456.64	94	26	52.57533	17	-1.14	30.065	6	45	3	12.83806	alpha/bet	55.34228
2e0q	P 1 21 1	455.26	95	26	65.11773	33	-2.4	31.07	5	55	1	4.915038	alpha	81.59403
3h31	P 21 21 21	454.8	90	28	61.14556	23	0.95	27.445	5	50	1	5.35785	alpha	53.72361
1lwb	P 1 21 1	454.265	95	30	64.05512	23	0.42	28.875	3	26	0	8.061088	alpha	74.58862
1byp	P 43 21 2	454.21	92	28	63.5565	22	-1.42	25.74	4	39	0	6.61313	beta	55.42811
1xww	P 1 21 1	453.19	95	24	48.86692	31	-0.4	37.475	6	60	1	9.271762	alpha	84.37741
2pne	P 1 21 1	452.93	91	40	68.12863	8	-4.14	22.845	6	19	1	7.145961	coil	15.34012
2wcj	P 32 2 1	452.59	96	34	47.25469	8	-4.98	28.08	4	42	5	6.047681	alpha	17.37776
1qzm	P 43 21 2	450.98	92	25	50.52885	25	-1.3	30.84	3	41	5	7.760588	alpha	76.99898
3ipz	P 3 2 1	450.3	96	24	62.22518	28	1	31.27	4	38	0	4.811792	beta	67.21519
2cwy	P 21 21 21	449.195	101	34	52.2045	17	-0.61	29.335	5	50	4	6.55172	alpha	50.63725
2ci1	P 1 21 1	448.875	90	26	55.05207	16	0.04	26.755	8	44	0	15.7594	alpha	46.50404

2w86	P 21 21 21	448.825	99	34	68.33287	25	-1.87	26.62	6	61	5	13.69324	beta	52.5628
2wut	P 41 21 2	448.8	92	22	54.95766	30	-0.52	26.22	6	53	4	7.550134	beta	73.51604
1x3o	P 43 21 2	448.73	88	25	57.42986	10	-2.3	28.01	4	33	0	5.490495	alpha	41.613
1vgj	P 21 21 21	448.095	90	25	60.40125	8	-3.07	21.4	5	37	1	12.11127	beta	29.60533
2uux	P 21 21 2	447.78	82	28	65.28876	29	0.88	29.02	6	53	6	3.097771	coil	81.62937
1rra	P 31 2 1	447.76	104	34	66.35698	23	-0.96	22.31	6	36	4	7.643045	coil	52.69341
1vem	P 1 21 1	447.545	90	29	62.8205	17	0.56	27.645	5	49	1	19.1017	beta	42.77112
1iuz	P 43 3 2	447.28	100	30	55.52674	25	-1.53	28.785	5	66	1	5.380008	beta	67.27106
1pmy	P 21 21 21	446.71	93	30	61.8634	14	-0.6	28.255	5	24	1	6.338833	beta	46.5078
1c8x	P 1 21 1	446.61	95	29	45.85656	11	-2.55	18.55	8	47	0	12.5445	coil	44.87136
1e0c	P 21 21 2	446.22	96	26	72.48667	31	-0.88	30.17	4	43	2	12.90339	alpha	75.41347
1i2t	P 21 21 21	445.735	95	26	62.91182	21	1.1	29.58	4	37	3	4.474901	alpha	71.94634
2lhb	P 21 21 21	445.495	83	28	50.00056	22	-3.5	26.82	6	44	3	12.63651	alpha	42.85794
2ah5	P 21 21 21	444.96	97	27	60.46611	33	-0.72	32.04	3	67	1	8.434466	alpha	68.22299
2ra1	P 1 21 1	443.195	103	35	53.37605	32	-1.79	26.88	6	54	4	13.15899	alpha	77.05863
2ici	P 1 21 1	441.44	95	22	52.60738	15	-0.06	32.815	9	74	2	12.30149	coil	46.28828
1srv	C 2 2 21	440.9	96	30	63.89431	21	-1.42	30	4	29	4	6.514221	alpha	73.10274
3fp5	P 21 21 21	440.73	90	27	56.56184	17	-0.39	23.15	6	45	2	12.27536	alpha	34.79455
1m2k	P 1 21 1	440.2	93	26	65.12154	33	0.36	34.65	5	52	5	10.77208	coil	78.23716
1c7k	P 21 21 21	439.62	95	31	47.06565	8	-0.44	29.325	5	40	5	7.178063	beta	25.02502
1bqb	P 21 21 21	438.57	101	31	45.77034	15	-2.56	24.585	7	55	5	11.60476	coil	31.89913
1acf	C 1 2 1	438.5	105	30	67.49259	18	-1.84	33.3	3	19	0	5.072109	alpha	54.75827
1snb	P 1 21 1	437.885	99	29	48.52187	24	0.61	35.325	5	55	0	5.410667	coil	74.0103
2hpj	C 1 2 1	437.37	94	29	50.91113	20	0.99	28.77	5	43	3	6.705695	alpha	64.87185
1g5a	P 21 21 2	437.295	112	31	51.71566	25	1.45	25.895	8	54	2	18.84712	coil	73.77171
1s3g	P 31 2 1	436.375	93	29	69.22257	28	-1.33	31.465	7	31	0	15.55341	alpha	68.21885
1zlm	C 2 2 21	436.22	98	24	47.45541	35	-2.8	37.39	6	64	0	3.736922	beta	74.26069
2y88	P 43 21 2	435.75	98	29	60.78026	13	-1.68	27.105	7	38	2	8.953528	alpha	56.48308
1ws6	P 31 2 1	435.455	99	28	53.09159	14	-1.4	27.44	5	42	2	9.06027	coil	49.4345
1a8d	P 21 21 21	435.42	102	28	85.07648	12	-0.29	20.855	7	37	5	14.9907	alpha	103.4128
1ok0	P 21 21 21	434.49	98	32	56.10256	30	1.38	31.04	5	38	2	4.839283	beta	77.866
3q1n	P 21 21 21	434.365	91	30	56.39727	26	-2.53	29.935	7	45	4	8.787817	beta	62.03654
1bh6	P 21 21 21	434.015	87	31	43.98005	17	-1.69	23.86	6	50	2	14.03608	coil	54.07071
3g4p	P 41 21 2	433.7	102	32	63.17155	30	-3.42	30.695	5	43	2	9.159258	alpha	68.61771
1l9l	P 1 21 1	433.3	87	26	48.45604	14	-0.67	26.965	4	49	4	8.412186	alpha	44.06647
1kp6	P 63 2 2	433.115	88	29	54.90343	20	2.57	22.87	5	38	5	8.228761	beta	59.25909
1wd3	P 21 21 21	432.425	96	35	53.71567	8	-1.35	23.01	12	22	2	22.1891	coil	21.77141
3nds	P 43 21 2	431.725	90	26	47.35074	36	-0.24	32.47	4	52	3	6.543216	beta	69.45857
2ftb	P 43 21 2	431.16	91	30	63.97393	14	-2.52	25.62	8	18	2	9.072317	beta	45.51327
1lri	C 2 2 21	430.48	94	30	80.27318	19	-0.3	26	6	28	0	6.687581	alpha	77.02797
1e9m	P 21 21 21	430.2	81	26	69.99651	14	-0.54	25.105	6	34	4	5.938796	coil	44.85472
3ij8	P 21 21 21	429.92	101	28	43.75349	23	-3.25	28.81	7	55	3	22.38119	alpha	53.21804
1g33	P 21 21 2	428.655	86	25	58.77454	14	-2.13	28.985	4	43	2	5.267336	alpha	49.97026
1mba	P 21 21 21	428.315	90	30	63.58988	13	-2.91	27.43	5	12	0	8.2737	alpha	56.98843
1noa	P 21 21 21	427.175	96	29	62.15485	22	1.04	31.465	8	39	1	8.019243	coil	61.21496
2z2x	P 21 21 21	426.87	93	25	63.92579	20	1.1	31.61	6	41	0	18.31909	alpha	64.21861
2qhe	P 21 21 21	426.645	90	23	80.69355	22	1.66	29.01	4	29	2	10.18088	alpha	67.56906
1gh2	C 1 2 1	426.38	98	30	49.40194	20	-3.41	30.57	6	57	2	7.875885	alpha/bet	49.06304
2rkq	P 21 21 21	425.99	94	27	52.84396	20	-3.07	28.615	6	59	6	7.320594	coil	62.47799
2wfb	P 31 2 1	425.9	96	26	65.33224	15	-1.18	22.88	8	40	0	6.339516	coil	47.83752
2pwa	P 43 21 2	425.6	98	30	56.23355	29	-0.72	33.88	6	56	0	10.07899	alpha	72.60103
1vyf	P 21 21 2	425.07	76	24	61.70984	16	-1.62	24.63	4	52	4	15.19691	beta	55.77905
2ayh	P 21 21 21	424.835	91	29	54.1316	13	-0.9	23.34	8	18	1	9.970035	coil	56.81029
1wmd	C 2 2 21	424.75	104	36	54.86521	29	0.6	29.85	10	60	4	28.62889	coil	68.42613
1b8l	P 1 21 1	424.69	87	25	61.53312	21	-2.98	28.265	6	31	2	10.56947	alpha	51.05606
3nul	P 21 21 21	424.51	89	28	56.70891	27	-3.38	26.23	6	56	2	10.16848	coil	48.26506
1nq6	P 21 21 21	423.85	92	25	62.03256	23	-1.33	31.75	5	33	0	8.273257	coil	63.11785
1os8	C 2 2 21	423.63	103	34	55.15426	17	0.04	22.94	12	44	0	16.8738	coil	51.99585
2coq	P 21 21 2	422.965	96	30	46.82421	17	-2.21	28.79	4	46	4	8.609743	beta	50.81153
1mk0	P 21 21 21	422.535	93	22	68.15175	24	-2.27	31.51	6	40	0	7.180825	alpha	83.34576
1i76	P 21 21 21	420.31	101	32	48.79256	32	0.29	32.775	6	63	3	10.63946	alpha	66.34865
1gvd	P 21 21 21	420.19	89	22	61.46029	18	-1.57	26.835	4	32	3	4.345344	coil	57.7691
1crb	P 21 21 21	419.035	93	28	65.45038	18	1.31	29.4	7	32	2	7.144081	coil	35.62352
1s29	P 21 21 21	418.98	86	23	52.08124	20	-0.48	33.995	5	61	4	5.888396	alpha	69.86252
1iiz	C 2 2 21	418.41	94	24	45.37893	17	-1.52	20.09	6	52	0	11.68799	alpha	57.42454
16pk	P 21 21 21	417.65	95	31	59.63845	17	-3.17	29.25	5	32	0	19.98413	alpha	35.20771
1czn	P 21 21 21	417.58	97	26	52.44744	23	-2.31	32.515	6	38	0	8.745031	coil	76.27042
2vg9	I 2 3	417.58	104	34	68.6575	35	0.7	33.08	10	65	1	11.61423	beta	62.76642
1oew	P 1 21 1	417.35	84	30	60.46484	8	-1.21	18.765	8	31	2	16.6991	coil	33.32335
3a38	P 21 21 21	417.14	86	24	63.16704	35	-0.82	32.06	7	56	2	7.41926	alpha	67.86211
2cg7	P 21 21 2	416	100	32	61.84615	24	-0.48	29.15	5	39	1	11.91796	beta	59.69952
1nd7	P 1	415.21	92	30	53.98955	21	-0.2	29.09	4	36	1	9.567135	alpha	60.59464
1uas	P 21 21 21	413.72	91	29	41.86044	20	-1.84	29.25	6	34	3	12.04075	alpha	73.26574
1m6t	C 2 2 21	413.5	84	22	59.8815	33	-3.86	28.62	4	36	2	10.66776	alpha	63.67352
2bo1	P 21 21 21	412.47	96	30	52.44139	20	-1.29	30.135	7	49	2	8.362426	alpha	47.18283
1pq7	P 1	411.585	85	35	55.65801	18	0.42	29.54	6	27	1	10.29921	alpha	48.68982
1yu5	P 21 21 21	410.975	89	26	64.44674	16	-0.46	29.44	4	36	2	6.26588	alpha	55.68709
1kqw	I 4	410.69	92	32	51.43174	10	-2.11	23.52	7	26	2	11.93236	beta	56.00331
1dv8	C 1 2 1	410.38	104	34	54.72976	15	-4.46	28.92	6	40	2	8.100712	coil	56.63288
2a7b	P 21 21 21	409.145	87	26	49.89307	28	-0.36	30.465	5	49	1	7.226045	beta	65.4829
1qua	P 21 21 21	409.045	80	24	47.70869	29	0.74	32.465	4	39	1	9.62058	alpha	68.17098
1war	P 43 21 2	408.88	100	30	44.78331	9	-1.59	20.865	6	25	1	21.35375	coil	24.91195
2zzj	P 21 21 21	408.19	90	27	52.49394	19	-2.42	25.8	7	19	2	7.871334	beta	58.15552
1kte	P 1 21 1	407.89	103	38	45.25117	11	-3.32	20.53	7	13	1	9.498885	alpha	43.13908
2hyv	P 21 21 2	407.7	93	24	49.89821	40	-1.04	33.855	5	59	1	15.60429	alpha	73.08805
2pvb	P 21 21 21	404.99	93	25	68.35848	25	-2.43	31.57	4	33	2	9.883577	alpha	79.97975
2ce2	C 2 2 21	403.495	100	29	48.35624	20	-1.62	27.88	7	42	1	6.984275	coil	55.89165
3bn6	P 21 21 21	403.03	93	31	46.93943	18	-2.48	29.125	6	32	5	12.48572	coil	54.39918
1wdy	P 21 21 21	401.785	95	27	49.46302	11	-3.66	28.995	4	30	2	13.94402	alpha	53.10054
1pne	C 1 2 1	401.35	96	25	48.60097	8	-2.55	24.65	5	25	0	8.232515	alpha	40.80354
3kt9	C 1 2 1	400.81	87	27	55.90305	28	-1.48	27.605	6	39	4	5.422769	beta	65.93648
2fdn	P 43 21 2	400.595	81	29	66.18904	14	-0.08	27.65	5	31	1	4.355696	coil	23.05695
1ra6	P 65	399.67	96	24	60.62877	17	1.35	31.355	6	43	0	12.06713	beta	57.83897
1vbw	P 1	397.455	84	20	62.97317	23	-0.58	30.62	5	43	0	8.618888	beta	73.22087
1bxv	P 41	397.01	80	29	54.65354	25	-1.1	26.845	5	35	0	5.780711	coil	63.75028
2rct	P 21 21 21	396.485	97	24	64.90157	29	-1.7	34.545	6	33	1	5.303151	coil	66.74906
1fkq	P 21 21 21	395.97	87	28	51.72109	18	-1.77	24.575	6	27	3	10.08314	alpha	55.81862
1ag6	P 31 2 1	394.43	86	25	49.76929	23	-1.05	29.265	5	48	1	7.66676	beta	59.32865

2w5q	P 21 21 21	393.99	87	27	55.12957	22	-1.31	31.885	7	46	2	25.15038	alpha	54.98109
1d2p	P 21 21 21	392.555	88	28	43.14427	19	-4.5	26.09	6	47	1	16.17192	beta	45.07903
3k89	P 21 21 21	391.61	88	26	62.78696	26	-1.58	30.055	4	29	0	9.954087	alpha	46.71357
2g6f	P 21 21 2	391.465	79	25	55.46345	11	-1.15	23.185	5	42	2	5.000447	coil	32.86628
2i4a	P 21 21 21	390.425	92	29	65.42614	28	0.74	30.255	6	49	2	11.13402	coil	65.56573
1rro	P 21 21 21	390.42	91	28	55.90134	11	-5.98	24.51	5	33	1	5.81776	alpha	25.24461
1gci	P 21 21 21	389.41	86	28	50.27477	22	1.53	27.03	5	28	2	11.25837	coil	66.05249
1gnu	P 21 21 21	389.18	85	27	54.26152	32	2.33	28.5	5	35	2	14.98535	alpha/bet	73.72809
1mhn	P 65	389.125	73	25	64.67459	27	1.47	24.915	5	34	1	5.516222	beta	72.71571
2o9s	P 21 21 21	388.37	69	19	63.78711	22	0.49	25.31	6	34	3	6.343822	beta	76.40652
1b0y	P 21 21 21	388.25	77	28	52.69028	12	-2.63	26.145	7	29	0	6.623954	alpha	38.59111
1ppn	P 1 21 1	387.77	84	28	45.19818	14	-2.17	17	8	28	2	18.17314	coil	39.49893
1qrp	P 21 21 21	386.71	82	31	45.22769	13	-0.75	26.24	5	41	3	8.8322	beta	38.72023
1pvx	P 21 21 21	386.375	94	25	48.4594	22	0.37	31.855	7	35	3	10.77029	beta	64.62245
1m8u	P 1	386.325	88	29	36.41364	15	0.1	20.77	10	44	0	18.60804	beta	67.51958
1joi	P 21 21 21	386.17	85	30	57.99389	28	-2.85	27.34	6	37	3	11.9646	beta	60.1678
1uha	P 1 21 1	385.46	91	26	54.04971	20	-0.65	31.18	5	39	0	4.771883	coil	59.36414
2fma	P 21 21 21	384.04	87	26	63.75768	25	1.27	29.25	5	49	2	5.492579	beta	61.16941
2cyj	P 21 21 21	382.52	75	17	70.97407	15	-0.57	27.05	7	33	2	8.231909	alpha	55.84937
1tt8	P 1	381.925	77	29	61.01591	27	-0.3	24.095	4	24	1	12.30084	beta	55.82771
3hbe	P 21 21 2	381.58	90	31	55.11557	23	-1.23	27.745	7	38	1	8.906704	coil	58.82777
1bol	P 21 21 21	379.905	88	27	35.95899	8	-1.31	21.44	8	35	1	13.66328	alpha	26.37501
1x3k	P 21 21 2	379.46	80	26	59.50298	8	-1.22	16.95	6	16	0	17.48603	alpha	38.36241
2hbg	P 21 21 21	379.19	85	25	74.01039	16	-2.23	25.76	5	17	0	11.2681	alpha	70.65587
2h5c	P 32 2 1	378.54	78	25	49.90358	10	-1.02	23.21	9	32	1	11.64408	coil	39.36704
1bud	P 43 21 2	377.41	87	27	55.98951	11	0.9	23.51	5	53	2	12.46589	alpha	63.19387
3g5t	P 21 21 21	377.335	69	19	56.70956	20	-1.91	27.78	5	26	5	16.8153	alpha	72.09376
2b4l	P 21 21 21	375.515	83	27	46.82902	13	-5.85	32.78	6	39	0	10.192	coil	31.6006
1q2y	P 21 21 21	375.355	87	25	54.21534	16	-3.54	27.63	6	40	5	13.80192	beta	54.31525
1cxl	P 21 21 21	375.045	78	29	51.51115	12	-0.59	25.145	6	38	1	9.91681	beta	48.83814
3ab6	P 43 21 2	374.655	70	24	49.26132	20	-1.56	25.51	5	35	2	13.2602	alpha	46.1598
2eql	P 21 21 21	374.265	78	23	57.16671	18	-0.48	27.06	6	31	1	8.819286	alpha	39.69113
3gmv	C 1 2 1	374.2	83	29	56.94281	23	-1.36	29.88	6	42	2	7.007937	alpha	72.85542
1ucd	P 21 21 21	373.23	93	30	62.11317	24	-1.68	31.345	4	43	0	8.789486	alpha	63.21705
1yw9	C 2 2 21	372.76	77	21	56.14202	19	-1.7	26.575	4	54	1	9.597328	alpha	54.38083
1fus	P 21 21 21	371.915	79	27	53.53374	20	-0.2	21.805	7	41	2	10.70809	coil	65.6884
1ua7	P 21 21 21	371.665	86	27	48.26927	27	-0.43	30.86	4	32	3	13.44859	alpha	77.87255
1dix	P 21 21 21	369.78	89	27	45.45811	19	-0.99	29.255	9	44	1	6.434556	coil	60.63741
3fh2	P 21 21 21	368.425	80	23	52.13408	17	-1.06	24.485	4	42	2	12.52255	alpha	63.85832
2ayd	P 32 2 1	368.19	82	24	52.51772	23	0.52	28.02	4	55	2	7.543985	coil	72.06198
1bm8	P 41 21 2	367.31	92	28	54.50709	37	-2.23	31.46	4	48	4	7.67232	beta	80.24557
2vk3	P 41	366.67	86	25	50.00136	16	-1.5	26.17	8	65	1	9.913192	alpha	62.54261
1mjn	P 21 21 21	365.87	86	24	63.01009	35	-0.62	31.91	5	45	2	12.97898	coil	79.56651
1o8v	P 1 21 1	365.605	86	31	51.3546	9	-3.44	22.755	6	19	1	14.2623	beta	29.93121
2o04	P 1 21 1	364.035	71	24	48.77141	14	-2.08	25.775	4	48	3	10.55975	alpha	48.04895
2whl	P 21 21 21	363.135	92	27	48.84685	26	-2.06	30.965	8	39	2	15.00062	alpha	65.8777
2vim	P 1 21 1	361.32	83	23	54.03659	24	-1.49	27.625	6	30	3	8.257224	alpha	52.26115
2j44	P 21 21 2	361.23	86	34	35.71409	21	-4.58	22.42	8	49	4	21.80674	beta	63.19796
1a6m	P 1 21 1	360.325	89	23	67.22681	12	-3.07	26.41	5	18	0	9.572608	alpha	42.81829
2fba	P 21 21 21	359.615	79	26	53.66295	8	-2.58	18.23	7	43	3	25.29266	coil	43.78293
1n0r	P 21 21 21	359.3	80	27	41.29975	19	-2.32	21.37	8	50	0	9.891094	coil	53.85889
1a44	C 1 2 1	356.605	83	30	55.246	11	-4.39	23.78	8	21	1	13.46762	coil	25.45814
3dj9	P 1 21 1	355.04	75	25	61.91415	13	-2.29	24.405	7	13	1	12.08208	coil	48.65508
1ucs	P 21 21 21	354.165	78	25	55.91462	17	0.68	26.6	6	44	1	6.556266	coil	39.69336
1dpo	I 2 3	354.12	82	24	57.8335	27	2.42	26.93	8	53	6	22.57814	coil	72.60816
1qnx	P 21 21 21	354.025	84	21	58.23882	27	-1.93	30.52	7	63	3	13.82318	beta	79.58054
1gd6	P 41 21 2	352.845	81	21	43.67357	21	-1.24	26.83	6	28	1	15.38069	alpha	49.77256
1idk	P 21 21 21	352.515	81	29	51.26307	35	0.56	30.215	5	44	5	13.82494	coil	79.92993
1frd	P 65	351.22	70	22	53.11201	17	-1.14	23.785	6	30	2	10.86817	coil	43.66636
1z3q	P 21 21 21	350.405	79	24	39.57278	13	-1.09	24.785	7	35	2	14.88098	beta	55.79829
1ixh	P 21 21 21	350.255	84	29	41.23424	21	-5.96	27.845	9	44	1	18.8477	alpha	62.63865
1vcc	P 21 21 21	349.94	76	20	58.99869	21	1.76	21.67	5	35	3	10.55109	beta	67.15866
3ctk	P 21 21 21	349.875	71	22	54.75098	13	-0.03	23.395	5	20	0	15.06752	beta	30.92533
2cbp	P 21 21 21	349.295	72	20	55.05232	26	0.8	27.835	6	44	3	7.024492	coil	67.17388
1enh	P 65 2 2	348.865	82	25	48.12893	35	0.34	31.405	3	54	4	7.923179	alpha	76.65859
1aba	P 21 21 21	347.72	73	19	59.90596	34	-0.66	28.185	2	23	0	8.444294	alpha	91.75917
3k6i	P 21 21 21	347.46	74	24	36.47758	22	-2.77	26.43	5	57	5	9.557935	coil	58.6715
2qsk	P 21 21 21	346.585	70	22	61.30531	20	0.26	25.375	4	43	0	14.62628	beta	47.78337
1g66	P 21 21 21	345.51	83	27	52.75679	16	-1.06	29.745	5	42	1	11.97577	alpha	63.02423
1eb6	P 1 21 1	344.595	76	28	45.50995	9	-4.59	23.735	5	34	3	13.81947	alpha	26.45134
1a70	P 21 21 21	343.97	79	20	55.98599	29	0.1	27.94	4	26	2	6.073553	alpha	79.87761
2baa	P 1 21 1	340.815	78	25	66.51409	14	0.65	22.77	6	15	1	17.7457	coil	38.72482
3bwy	P 21 21 21	340.195	69	26	42.91509	12	-2.73	19.945	5	30	3	12.95654	alpha	31.4599
2ppn	P 1 21 1	339.695	74	27	45.84554	15	-2.41	24.745	7	40	3	6.517611	coil	55.96344
2x46	P 21 21 21	338.965	72	27	45.31294	10	-2.26	21.785	7	43	3	10.14594	beta	35.10392
1cuo	P 1 21 1	338.66	73	27	59.15372	21	-2.75	21.325	5	18	2	8.869515	beta	36.01252
2d58	C 1 2 1	338.495	73	17	81.92883	27	-2.04	31.885	3	26	0	8.165911	alpha	74.81942
1iwd	P 21 21 21	338.055	81	24	44.42324	11	-2.35	23.87	6	24	0	12.72905	coil	46.85184
2hzc	P 43 21 2	337.885	83	22	47.23205	27	-0.68	32.86	5	47	3	5.343889	alpha	74.71625
1bru	P 21 21 21	336.685	79	25	47.21179	22	-0.39	25.16	8	52	2	11.28726	coil	59.69378
1ops	P 1 21 1	334.68	77	26	56.48231	10	-1.75	27.015	6	32	3	4.820276	coil	40.13685
1gbs	P 21 21 2	334.01	82	20	49.58235	17	-0.84	23.37	4	48	4	9.053621	coil	61.56403
1sup	C 1 2 1	333.79	77	26	60.09317	13	0.22	24.02	7	22	1	10.10102	beta	49.24653
2fcr	P 21 21 21	333.51	74	23	59.66088	19	-3.02	23.615	5	16	1	12.18404	alpha	67.19289
1op0	P 21 21 21	332.98	68	22	58.76479	9	-1.95	16.095	9	43	2	14.28125	beta	32.17611
3qgv	P 41 21 2	330	83	32	45.92424	6	-3.18	16.93	10	36	3	29.54658	alpha	34.16667
1i5g	P 21 21 21	329.17	73	21	59.87028	15	-1.88	24.8	4	15	1	11.47316	alpha	55.42273
2qpz	P 61	328.31	79	24	56.19384	27	0.01	23.21	6	24	1	10.37242	beta	69.85319
3n5a	P 21 21 21	327.93	70	22	67.47782	21	-2.26	24.535	5	34	1	19.86323	coil	54.71899
2y24	P 32 2 1	327.06	94	24	63.10157	6	-0.4	21.96	8	44	2	31.16401	coil	17.30264
3k6d	I 41 2 2	326.06	68	22	52.52408	12	0.44	20.41	4	20	0	12.73155	beta	35.06103
2mhr	P 21 21 21	323.62	61	21	70.89488	21	-0.16	21.545	4	16	0	12.22267	alpha	63.66726
1h0p	P 61	323.575	84	25	58.28633	18	-1.76	27.23	7	15	1	13.31954	coil	55.6378
2igd	P 21 21 21	322.445	68	20	58.01299	25	-0.73	22.765	4	31	5	8.708462	beta	85.83634
2fe5	P 1 21 1	320.985	65	20	55.93096	15	-1.39	22.07	5	33	2	15.35118	alpha	48.45554
1hg7	P 21 21 21	319.34	63	22	65.07328	25	1.56	24.505	5	31	2	6.351757	alpha/bet	59.05774
1uoy	C 2 2 21	317.98	72	24	50.22328	19	-0.66	23.94	6	42	4	4.670105	coil	51.92779
3pvh	P 21 21 21	317.38	72	28	38.17033	12	-3.41	22.515	4	60	4	15.03639	alpha	71.86181

1sca	P 21 21 21	315.19	72	25	62.32431	28	1.45	27.195	4	37	1	17.38951	beta	48.58339
1mxg	P 21 21 21	313.2	84	25	52.98691	19	1.12	27.34	6	37	1	24.49352	alpha	55.25543
3kiv	P 21 21 21	313.18	77	26	45.28865	18	0.26	26.8	6	34	1	8.07162	coil	73.41305
1hpi	C 2 2 21	309.63	70	20	49.61083	14	-0.47	27.79	4	19	1	3.335433	beta	58.86542
1yna	P 1 21 1	308.41	72	22	48.08048	29	-0.35	25.94	8	45	2	13.04432	beta	60.93512
1jo8	P 21 21 21	307.785	66	20	59.38074	20	-0.33	23.715	6	27	0	8.936823	alpha	72.03243
1zma	P 1 21 1	306.575	72	22	51.77852	10	-2.08	22.915	5	23	2	10.88761	alpha	36.54081
2w9q	P 21 21 21	301.935	64	18	73.18959	27	-0.58	25.325	6	16	1	8.416944	coil	78.99217
1mjc	P 21 21 21	301.34	64	20	54.16141	27	-0.46	25.3	3	33	2	9.542377	beta	44.64226
1emy	P 21 21 21	296.125	66	24	54.91093	6	-3.34	20.065	5	22	1	8.411144	alpha	30.10891
2w39	P 21 21 21	295.12	69	26	66.46279	19	-1.9	20.89	7	21	0	18.412	alpha	70.55604
2sn3	P 21 21 21	294.205	58	16	59.96669	21	-1.47	25.31	3	38	4	7.341819	alpha	66.54374
2ov0	P 1 21 1	293.985	70	19	66.91498	20	0.46	25.815	3	24	1	10.09106	beta	59.97075
2mcm	P 1 21 1	291.725	63	21	47.91499	11	-2.21	23.31	6	23	3	11.17576	beta	35.40321
1b02	P 21 21 21	290.735	60	15	64.20796	12	-1	20.555	3	22	2	18.03962	beta	44.80197
1thm	P 21 21 21	289.33	68	26	50.60657	10	-0.16	21.11	6	27	1	22.16327	beta	50.00691
2acg	C 1 2 1	287.44	68	24	81.5231	29	-0.08	22.47	2	27	2	8.289556	coil	55.10715
2vn4	P 21 21 21	281.415	64	24	62.01517	20	-0.7	23.035	6	25	0	20.77181	coil	59.70364
2dsx	P 1 21 1	280.725	62	19	51.16217	10	-2.93	21.155	5	32	0	6.997061	alpha	31.30822
1iqz	C 1 2 1	280.625	64	21	65.78174	20	0.76	25.355	6	21	1	11.41335	coil	71.34967
1seg	I 4 2 2	270.38	70	19	63.93779	13	2.08	21.06	5	17	0	8.15101	coil	28.95554
1bf2	P 21 21 21	270.365	62	19	62.04575	24	-0.12	25.775	5	32	2	16.29046	alpha	51.38979
1plc	P 21 21 21	268.145	58	17	48.86908	24	-1.54	23.66	5	31	0	9.741931	alpha	61.95901
1rb9	P 1 21 1	265.9	58	18	50.72396	31	-1.33	25.135	3	19	1	4.607446	coil	57.73223
1brf	P 21 21 21	256.245	57	18	74.21413	26	-0.52	23.225	4	20	0	6.769888	beta	60.6607
1npi	P 1 21 1	255.665	69	19	62.12231	29	0.45	26.48	5	26	0	6.243991	coil	65.42155
2ckx	P 21 21 21	237.96	60	17	61.49353	40	0.22	26.535	4	44	0	9.403345	alpha	93.37494
2cfe	P 41 21 2	219.97	47	17	70.13911	13	-0.2	20.325	4	18	1	19.53162	beta	48.84302
2b4z	P 21 21 21	205.09	47	17	59.42757	9	-2.14	17.43	4	10	0	21.88673	alpha	50.81428
1jix	P 1 21 1	196.015	40	11	63.05895	22	-0.27	18.375	3	19	0	28.80581	alpha	84.8634

large crystal packing contacts

		IA	NA	NR	fnp	fbu	Rp	LD	Nbseg	Nnbc	HB	Igap	SSI	fcore
1muw	I 2 2 2	4563.53	956	242	67.26657	40	8.42	58.91	18	606	34	2.12771	alpha	77.87349
1x0m	C 2 2 21	3484.62	704	170	63.14749	39	6.7	47.78	20	390	18	2.866884	alpha	84.77395
2cyy	P 61 2 2	3193.26	624	144	64.9161	35	2.66	57.97	8	391	30	2.580983	beta	85.28839
2f82	P 61 2 2	3074.37	636	162	58.20965	41	-2.38	55.72	12	471	23	2.637978	coil	83.33935
1nhs	I 2 2 2	2885.55	574	162	68.8361	40	6.82	50.72	14	238	6	3.405936	alpha	73.92005
2pes	I 2 2 2	2849.18	574	150	61.87535	37	6.3	44.84	18	352	33	2.327642	beta	74.31191
1cib	P 32 2 1	2824.63	558	162	56.13302	25	-6.6	48.69	18	337	13	4.313397	alpha	77.23277
3o04	P 43 21 2	2550.62	546	150	70.80631	32	-0.64	45.41	18	294	6	3.294787	alpha	71.35206
3fk5	P 21 21 2	2309.38	484	114	56.09428	37	1.88	47.64	12	254	11	3.501589	coil	76.57467
1fl2	P 32 2 1	2238.6	466	112	67.4015	40	3.56	49.71	16	278	6	3.450983	alpha	84.3889
1t71	P 43 21 2	2207.9	440	116	71.62326	34	7.02	45.95	12	251	7	3.087776	alpha	76.65972
1wgb	P 41 21 2	2183.27	472	110	73.3693	38	6.26	56.81	12	311	12	2.012692	beta	81.69306
1n70	H 3	2124.67	433	121	60.51693	45	4.18	48.99	13	300	20	4.082399	beta	81.90754
1cs3	I 2 2 2	2068.63	388	94	64.67324	36	8.46	42.06	8	184	14	2.396755	alpha	85.88824
1dus	P 21 21 2	2045.57	402	92	54.58479	20	-8.56	48.13	14	258	13	3.159569	coil	68.07394
1wwb	P 64 2 2	1971.61	428	96	71.1657	47	5.18	45.21	6	346	10	1.193121	beta	93.25982
1sk4	P 32 2 1	1947.71	394	100	72.64737	51	12.86	46.58	10	271	9	2.944032	coil	83.93447
2czr	P 43 21 2	1904.06	406	92	58.39522	33	-1.16	44.65	12	239	11	5.583464	alpha/bet	80.35566
1q33	I 2 2 2	1803.89	362	104	47.45023	22	-7.5	36.77	16	257	10	7.465117	coil	66.10714
3au2	P 1 21 1	1780.41	378	110	51.39855	16	-5.48	30.72	21	172	7	9.965328	alpha	49.39789
1qr0	P 43 21 2	1692.83	334	92	65.75616	32	5.46	44.37	8	228	6	2.765263	beta	72.24057
3fg2	P 32 2 1	1650.68	334	112	55.03005	13	1.88	38.84	18	167	8	7.601867	coil	51.73807
1xoe	I 4 3 2	1596.515	342	94	59.98847	33	0.65	44.545	11	268	7	3.259756	beta	73.77757
1n5d	P 43 21 2	1573.43	332	90	58.85867	16	1.04	48.53	14	176	7	6.878997	coil	49.93168
3c8y	P 42 21 2	1526.79	314	92	53.40093	30	-1.74	37.87	16	163	9	12.75028	coil	64.76071
1dot	P 21 21 21	1522.85	328	94	52.87454	19	-4.45	36.965	15	154	1	9.673881	coil	61.70207
1n81	C 2 2 21	1516.4	320	74	57.37734	38	4.28	45.05	6	331	2	4.306661	alpha	81.52334
1i5p	P 43 21 2	1509.935	329	98	48.01134	31	0.43	40.39	14	284	7	7.731538	beta	78.90803
2p26	P 1 21 1	1490.9	306	87	58.27889	25	-1.1	39.475	13	134	5	7.483902	coil	64.44295
1bs1	C 1 2 1	1488.08	298	80	63.28625	35	1.96	41.85	10	177	12	3.030079	alpha	81.38339
1svb	P 61 2 2	1457.19	354	98	67.29596	26	3.16	31.5	18	139	4	9.245877	beta	66.31531
2cw8	C 2 2 21	1451.28	342	98	51.6985	25	-10.52	34.74	20	142	0	11.94161	alpha	46.12411
1ew0	C 1 2 1	1437.49	294	82	67.34377	33	0.98	43.69	6	119	2	5.31082	alpha/Bet	72.91877
1wq3	P 31 2 1	1434.06	276	78	71.02632	58	11.76	49.13	10	175	8	2.39582	alpha	85.99849
2igx	P 21 21 2	1425.1	274	84	76.77988	23	6.98	36.82	12	104	0	5.657757	beta	58.03242
3kyf	C 1 2 1	1419.07	288	78	49.1512	27	-2.46	40.97	16	187	12	5.253701	alpha	80.31669
2r1n	P 31 2 1	1409.135	298	76	59.77036	42	5.66	44.47	10	251	8	3.812977	alpha	72.20529
2ywr	P 41 21 2	1405.67	254	68	77.48049	37	5.72	43.84	10	160	4	2.230516	beta	63.53127
1aj2	C 1 21 1	1384.42	290	78	76.89863	51	4.24	45.52	6	185	4	2.069726	alpha	90.19589
2is9	I 4	1353.47	254	74	61.21451	24	8	30.82	16	118	5	6.273874	alpha	63.72509
3edy	P 21 21 21	1352.38	295	78	57.87611	28	-0.14	39.045	12	143	5	5.919564	coil	81.16469
2yl6	I 2 2 2	1344.4	278	82	52.78414	17	-8.64	29.17	14	135	5	8.532892	alpha	54.27923
1vip	I 41 3 2	1344.38	274	72	70.51503	30	3.14	46.69	12	115	4	3.981575	alpha	82.6195
3n6t	C 2 2 21	1338.79	274	76	73.92347	29	8.3	40.18	10	107	6	5.299001	alpha/bet	67.05831
1dlc	C 2 2 21	1337.22	266	84	51.22194	12	-0.92	30.84	18	93	11	10.95622	alpha/bet	46.9459
1qba	P 21 21 2	1305	262	82	57.67969	19	-9.1	28.47	16	115	4	15.91293	alpha	47.38161
2zu5	C 1 2 1	1229.3	250	80	53.41658	34	0.24	37.15	14	175	12	11.32779	beta	75.90417
3kk8	P 65 2 2	1228.775	254	73	56.19987	24	0.24	37.985	11	147	7	7.580721	coil	72.03027
1wol	P 31 2 1	1159.03	256	68	60.30474	24	-1.6	37.2	8	160	3	3.503033	alpha	60.7439
1edq	C 2 2 21	1156.11	256	68	63.26042	29	-2.3	37.81	14	148	2	6.784389	coil	79.92838
1pll	I 21 3	1141.605	235	62	69.78333	43	5.41	40.875	8	140	7	2.566124	alpha/bet	77.26359
2ovu	I 2 2 2	1140.03	236	72	66.90701	25	1.32	33.24	10	92	2	3.854504	beta	66.97631
2p5y	P 41 3 2	1134.145	246	68	73.55144	50	0.6	47.4	6	106	0	3.19304	alpha	81.32205
1omr	I 4	1129.59	243	64	69.64607	41	-0.53	41.59	10	120	1	6.14592	alpha	69.79745
3acp	P 64	1125.96	244	68	58.23297	28	-0.58	40.64	10	118	4	7.283785	coil	53.48591
3cz7	C 1 2 1	1125.5	186	54	45.66148	12	-2.12	25.96	12	116	0	8.513212	alpha	38.17592
2eyi	P 21 21 2	1118.37	246	66	40.99001	16	-8.16	39.43	12	150	10	7.4328	alpha	74.09265
2azp	I 2 2 2	1111.27	244	76	60.31837	33	3.2	36.92	16	145	6	7.814366	beta	70.57871
2iwx	P 43 2 2	1101.14	244	58	62.20462	32	-5.54	32.57	8	158	8	5.094039	beta	76.74955
2pfz	P 41 21 2	1091.52	260	68	53.35495	14	-6.3	36.94	10	106	2	6.400478	alpha	39.64563
1af7	P 1 21 1	1090.73	219	68	60.68642	21	1.15	28.04	11	66	4	8.218349	alpha	64.96521
1ncx	P 32 2 1	1089.305	227	62	66.90459	37	2.04	36.08	8	96	4	5.731866	alpha	80.96676
3cu9	C 2 2 21	1081.76	230	66	58.96964	24	-1.44	38.14	10	68	4	5.618964	beta	53.76054

1pi1	P 32 2 1	1075.49	200	62	59.54495	23	3.14	36.06	10	68	4	6.627677	alpha	67.65195
2z00	P 65 2 2	1068.14	236	58	56.23607	42	-3.18	39.93	10	164	18	4.837493	alpha	77.26702
2xy2	P 31 2 1	1062.64	224	72	59.21855	26	0.26	35.46	10	88	2	7.628877	beta	63.29048
1kfw	P 32 2 1	1056.45	234	76	62.05878	22	-4.64	32.07	18	119	6	10.32515	beta	64.4252
3go5	P 32 2 1	1048.18	244	60	55.92837	21	-1.66	33.56	14	125	4	10.14902	beta	58.65214
1p3c	C 2 2 21	1042.69	244	80	57.72761	20	1.72	31.48	14	132	6	6.350641	beta	54.99238
1sdj	C 2 2 21	1020.985	184	58	67.96966	43	6.7	38.52	10	131	8	6.941826	beta	77.83072
3m66	C 1 2 1	1013.3	228	76	43.083	3	-10.66	23.32	16	53	6	13.20623	alpha	10.7816
1uok	P 62	1011.835	228	69	55.56192	11	-4.92	28.395	16	57	0	14.64295	alpha	37.62076
1gpi	C 1 2 1	1009.85	220	72	54.17339	16	-3.32	37.22	10	95	8	8.328465	beta	52.74942
1z1b	C 2 2 21	998.23	210	58	74.52491	28	1.24	39.41	10	117	6	4.273564	alpha	61.23739
2ggo	H 3	973.825	211	60	61.68665	28	-0.16	37.025	18	99	6	4.872795	beta	57.79221
3e91	P 32 2 1	967.4	216	58	58.54249	31	-0.36	35.93	8	84	8	12.67805	alpha/bet	79.83047
2ill	I 2 2 2	964.53	210	58	57.39687	15	-0.92	28.42	8	86	4	6.66931	beta	41.04175
1i60	P 21 21 2	963.68	216	60	67.89806	32	-3.6	41.38	8	132	7	5.736603	coil	72.29059
3gjw	P 3 2 1	957.31	218	64	63.84766	16	-4.64	27.38	10	77	3	14.43693	alpha	54.50272
1qts	P 1 21 1	956.32	198	57	55.50809	33	0.13	37.41	10	95	4	5.953677	coil	68.52361
2ya0	P 1 21 1	950.39	216	68	50.97855	15	-4.21	29.175	21	96	2	13.84958	coil	50.90016
1ei9	P 41 2 2	949.67	174	62	62.11421	11	7.8	18.05	12	59	6	7.907347	alpha	28.26561
1iow	P 21 21 2	945.36	184	58	80.0256	45	5.66	36.39	10	80	0	9.507087	alpha	87.78349
3crv	P 21 21 21	945	221	63	52.32804	14	-4.34	31.925	12	79	0	12.01071	coil	51.32063
1qwz	C 1 2 1	938.6	206	56	49.36395	15	-5.48	31.66	11	89	1	8.356595	alpha	60.18485
2d48	P 41 21 2	923.98	194	50	51.33661	35	-4.84	51.53	6	172	8	3.506569	alpha	84.24641
3p7k	P 63 2 2	923.745	157	43	78.29812	23	4.43	23.67	2	52	4	2.90827	alpha	58.51507
3o8z	P 32 1 2	920.6	182	44	71.35781	41	0.18	39.79	4	85	3	2.038345	alpha	92.27352
3lig	P 21 21 2	920.38	190	56	67.34827	22	0.74	34.32	12	158	4	5.922141	coil	47.86284
1r1h	P 32 2 1	919.35	174	62	68.78664	17	-3.7	21.36	12	57	0	18.88766	alpha	41.59678
1yfq	P 21 21 21	918.875	195	55	60.88532	19	0.96	35.34	16	83	2	9.964761	beta	54.23861
3fhg	P 6	913.45	202	52	55.53999	33	0.28	43.84	8	108	0	8.69752	alpha	82.1074

weak transient complexes

	IA	NA	NR	fnp	fbu	Rp	LD	Nbseg	Nnbc	HB	Igap	SSI	fcore
1S1Q A:B	643.85	129	36	56.8	26	1.46	32.4	6	81	5	4.48707	beta	67.92964
2OOB A:B	404	82	25	73	34	1.34	32.06	4	37	3	5.471832	alpha	64.27228
1GCQ B:C	603.8	124	36	55.5	40	3.19	37.26	6	98	4	3.806509	beta	72.02219
1AK4 A:D	514.35	111	30	64.5	31	2.28	34.6	7	65	4	7.952756	beta	66.06008
2AQ3 A:B	552.65	116	34	55.4	22	0.15	31.8	7	58	1	8.427576	beta	64.3599
1GLA G:F	652.05	130	38	64	23	-0.7	34.3	8	42	2	5.709102	alpha	53.66766
2PCB A:B	514.85	110	28	54.6	19	-3.61	28.8	6	31	1	10.65893	alpha	47.24871
1QA9 A:B	676.3	134	38	43.1	16	-7.76	32	5	79	6	5.309774	beta	41.33003
1Z0K A:B	893.6	181	50	58	35	1.25	38.1	8	104	7	3.897717	alpha/bet	71.74743
2FJU A:B	625.55	125	33	64.6	23	1.5	34.5	8	70	2	10.48837	beta	69.55479
1KLU A:D	627	135	37	61.8	26	-0.72	37.2	8	89	2	6.986842	alpha	81.77352
1EWY A:C	750.95	157	46	49	13	-1.06	32.6	12	62	0	9.932419	beta	47.68826
1HE8 A:B	652.45	138	39	52.5	19	-2.89	28.3	6	67	1	10.67668	beta	60.6192
1MQ8 A:B	626.45	127	40	54	35	-0.51	35.5	7	75	1	6.863676	beta	68.48751
1E96 A:B	589.35	123	38	60.9	28	2.8	32.4	6	72	5	9.334436	coil	78.65445
1F6M A:C	910.7	192	56	56.6	24	1.19	36.1	9	103	4	6.403865	coil	73.46602
1LFD A:B	593.15	124	34	50.4	31	1.02	34.4	7	68	4	6.958813	beta	61.96746
2PCC A:B	570.45	106	33	53.3	9	-2.85	23.4	9	25	0	9.152634	alpha	23.98633
1US7 A:B	547.55	123	38	54.8	34	-1.05	32.8	4	77	4	11.30445	alpha	81.58524
1FFW A:B	583.15	106	30	65.2	25	-0.97	29	5	58	2	4.138078	alpha	61.60851
1ZM4 A:B	777.05	172	53	52.4	13	-4.3	33.5	9	72	2	11.78785	alpha	48.8096
1J2J A:B	604.85	119	32	72.8	29	2.79	34.08	6	58	2	4.921468	alpha	85.96016
1AY7 A:B	616.635	134	37	54.14	34	1.05	42.21	6	119	8	4.690571	coil	66.4899
1F3V A:B	741.7	160	49	55.042	26	1.16	36.065	7	84	8	5.93821	beta	72.76999
1J7D A:B	773.875	165	40	67.9	38	1.43	40.695	6	83	3	6.266994	alpha	73.37748
2G45 A:B	492.02	112	28	59.64	34	1.32	34.8	7	82	2	4.842791	coil	84.55246
1USU A:B	707.565	132	41	45.17	11	-2.9	25.175	9	73	3	11.69575	alpha	37.23827
1UUG A:B	984.465	209	62	66.8	30	4.47	40.835	10	79	7	4.641861	coil	77.42022
1WRD A:B	544.965	116	36	63.4	16	0.23	29.795	5	37	1	5.059719	alpha	47.21037
2C7M A:B	705.975	154	38	67.8	33	1.09	36.255	4	66	2	3.795814	alpha	87.8381
2B11 A:B	565.81	116	33	56.12	11	-1.9	25	9	33	3	10.19401	coil	36.27808
1X1Y A:D	738.405	170	43	56.4	28	0.61	46.22	8	120	9	3.903346	coil	67.3912
2IYB A:E	757.955	151	46	51.42	30	3.22	32.665	11	100	4	3.709152	beta	72.156
2XGY A:B	764.87	174	47	58.36	34	0.31	41.545	7	117	6	3.538837	alpha	80.38556
2PTT A:B	727.435	163	46	40.18	25	-3.78	33.78	6	110	10	4.29626	beta	67.17026
2V3B A:B	576.85	119	40	59.03	19	1.96	29.39	10	54	1	9.700529	alpha	52.39577
2WO3 A:B	975.93	211	60	62.3	32	4.81	42.23	8	95	2	5.408687	beta	66.63183
2V8S E:V	666.425	141	37	51.86	26	-1.15	38.49	5	117	5	5.266909	alpha	73.27981
2YVJ A:B	655.185	153	46	58.5	19	-0.94	34.265	8	68	0	10.35909	alpha	51.79529
3BN3 A:B	772.335	155	43	53.6	26	-0.2	37.48	8	72	2	6.633456	alpha	52.91421
3F1P A:B	760.915	206	61	60.6	34	2.88	37.87	8	86	5	6.981397	beta	89.60331
3GC3 A:B	973.45	228	62	58.32	32	-2.43	35.37	9	96	4	8.393723	coil	73.80451
3CRK A:C	458.9	93	32	62.1	18	-1.19	24.105	6	51	2	10.06836	alpha	39.94116
3KUD A:B	522.78	111	29	50.8	34	-1.52	29.85	4	73	4	6.991698	beta	76.64218
3MA2 A:B	823.145	170	46	63.6	44	3.89	47.81	9	94	6	6.178887	beta	78.91016
3ONL A:C	671.78	137	35	46.4	26	-1.91	38.36	5	84	3	5.08927	alpha	61.51121
3OKY A:B	1031.255	208	72	58.9	15	-0.94	25.93	14	70	3	10.15195	coil	45.80584
3GJ7 A:B	450.06	90	31	66.97	29	-0.13	30.02	6	41	0	4.154446	beta	50.54215
3HCT A:B	553.91	113	33	63.73	27	0.08	30.805	7	48	3	7.41523	coil	62.67896
3RTO A:C	974.13	209	60	62.8	31	-0.32	42.945	10	104	4	6.128925	coil	72.82447
3FF7 A:C	537.64	112	34	60.22	39	2.53	34.615	5	66	4	5.77524	beta	74.51362
2WWK O:T	582.275	129	40	63.24	33	0.7	33.755	5	68	6	6.306299	beta	68.7785
5CRO A:C	648	130	38	73.6	35	2.88	30.1	7	64	5	4.203117	beta	80.3642
1BI2 A:B	785.4	159	42	75.9	47	4.04	37.39	4	73	3	4.787051	alpha	81.84874
1NFK A:B	684.2	141	35	62.8	28	1.93	37.24	6	76	1	7.483002	beta	79.17129
1RAM A:B	656.2	130	35	61.8	29	1.41	33.08	6	71	0	8.604648	beta	72.32627
1A15 A:B	740.2	160	40	63.6	39	2.05	38.74	5	111	7	6.625061	beta	84.7271
1DOM A:B	869.5	181	46	62.6	35	2.86	42.01	6	184	1	4.611271	beta	77.26394
1CNT 2:3	741.6	150	43	66.3	25	2	30.36	5	69	1	6.908374	alpha	68.27333
1BBH A:B	770.3	171	46	80.7	46	1.4	43.42	6	70	0	9.038842	alpha	83.58756
1BEB A:B	534.4	109	33	52.6	28	2.42	31.59	4	67	8	10.46476	beta	74.92234
2LYN A:B	889.6	176	43	72.4	30	7.28	38.12	4	76	0	5.110297	alpha	71.81374
1EDH A:B	901.6	191	61	57.9	13	-3.45	32.2	10	59	6	8.358884	coil	44.97116

1A78 A:B	510.4	104	28	57.8	32	1.46	29.71	4	69	7	6.764538	beta	91.04428
1TRZ B:D	624.6	136	33	71.5	29	0.3	37.65	2	79	4	2.004675	alpha	71.75712
1SCF A:B	829.6	177	42	62.8	32	-1.15	45.16	4	130	6	2.953532	coil	73.38175
1CMI A:B	818	186	48	63.4	48	0.92	40.04	6	127	11	3.399756	beta	99.7335
1BFS	683.9	130	32	64.5	34	2.8	36.22	6	119	3	3.237315	beta	91.40225
1A45	558.2	130	32	48.5	48	2.66	39.38	6	69	2	4.317001	beta	87.77678
1B8E	470.1	94	28	51.1	21	2.82	28.09	4	53	2	12.15458	beta	79.78515
1CBI A:B	556.3	104	37	73.3	23	0.78	29.02	6	27	0	8.790886	alpha	44.0329
1CXQ	795.4	176	42	65	23	3.14	33.64	6	93	4	7.616445	alpha	61.51873
1DOK A:B	900	185	47	55.6	42	2.22	38.75	6	157	7	2.4675	coil	86.69167
1DTO	984.8	202	58	56.4	24	-1.14	34.97	10	84	4	6.203036	alpha	65.61637
1EAJ A:B	677.7	133	43	64.9	30	-2.71	32.64	6	92	3	6.573705	beta	64.99779
1EEQ A:B	686.5	162	39	64	28	0.51	32.03	6	88	4	5.407866	beta	69.61034
1ERV	515.2	120	32	59.2	40	-0.52	38.03	4	51	2	3.052698	alpha	91.09278
1EXZ CD	852.63	182	43	61.3	38	-1.57	43.82	4	127	8	2.46604	alpha	85.36176
1GML AD	801.4	145	43	56	13	4.19	29.62	8	76	4	4.337721	beta	45.09109
1H65 BC	1240.6	262	75	60.69	15	0.18	35.275	12	149	4	5.625907	coil	52.36176
1HGX A:B	1048.4	236	63	65.2	31	3.28	40.52	8	94	5	3.933852	coil	77.99981
1IHK	1214.1	233	60	69.6	24	4.9	36.89	8	125	2	4.36156	coil	68.97125
1II5	1010.3	192	60	61.5	23	1.44	35.21	8	95	5	4.583292	alpha	62.86747
1ITV A:B	644.6	140	42	53.3	29	0.37	32.1	6	78	5	6.487155	beta	67.40537
1JR9	855.1	170	42	70.2	28	2.12	39.41	10	109	2	5.000725	alpha	75.89054
1LWJ A:B	1310.9	294	74	70.7	43	3.64	46	18	166	1	7.659333	coil	83.39614
1NEU	477.8	96	24	50.9	21	0.6	20.75	4	62	4	10.39058	beta	77.53453
1NRV A:B	399.9	77	22	62.9	30	1.58	27.09	4	46	4	7.249612	coil	59.30483
1O7Z A:B	491.7	102	32	66.7	25	0.1	28.74	6	35	5	7.74938	beta	56.07688
1P9Y A:B	264.3	54	19	71.1	20	1.12	17.74	4	6	0	11.30106	coil	48.23496
1PY9	911.35	206	62	60.1	34	1.56	40.78	10	140	4	3.86994	beta	64.43957
1SJ1 A:B	367.1	77	20	52.1	38	1.12	30.85	6	94	4	6.702179	coil	97.18469
1TIP A:B	931.1	202	62	48.7	25	-0.15	33.59	14	82	4	6.332424	alpha	56.09548
1UQ5	914	196	60	69.5	33	5.58	38.35	14	106	2	3.578085	beta	88.00875
1ZOP A:B	1082.2	203	48	58.7	35	1.75	46.17	10	140	9	3.305766	alpha	79.2871
2DTR	874.7	170	42	78.3	44	5.46	36.61	4	66	5	2.75108	alpha	85.57448
3BRI	840.2	184	50	70.5	49	1.7	40.37	6	119	10	3.101047	beta	82.54582
3IL8	924.7	190	54	56.2	29	-0.6	36.69	6	108	14	3.226452	alpha/bet	62.15097
3SDH A:B	886.1	178	53	54.9	35	-3.45	34.7	6	70	5	4.707708	alpha	76.91908
1DV8	410.38	104	34	54.7	15	-4.46	28.92	6	40	2	8.100712	coil	56.63288
1E87	869.31	171	48	48.39	17	-0.06	34.79	8	103	9	3.400973	coil	58.47224
1OAL	730.46	160	42	70.69	30	2.14	33.28	12	96	2	5.659954	beta	79.86337
1COW	796.4	153	42	77.5	44	5.1	37.3	4	91	2	2.483363	alpha	75.70128

permanent homodimers

	IA	NA	NR	fnp	fbu	Rp	LD	Nbseg	Nnbc	HB	Igap	SSI	fcore
1a3c	987.46	210	52	62	44	0.06	46.08	8	126	4	5.109696	coil	90.49177
1a4i	1337.75	272	73	59	37	-2.47	46.385	8	152	6	3.882728	alpha	75.06859
1a4u	2465.035	470	125	69	43	9.21	48.785	15	212	9	1.99211	alpha	81.74367
1aa7	1089.1	215	52	61	33	-0.96	43.75	5	121	2	2.503902	alpha	85.11707
1ad3	3901.4	773	220	64	44	3.78	46.39	20	500	15	4.123807	alpha	80.76285
1aq6	2217.18	447	107	66	62	5.93	53.21	10	302	13	1.864697	alpha	92.19504
1af5	835.6	194	48	68	43	-0.62	46.49	8	102	0	2.819232	alpha	76.20752
1amk	1476.78	306	78	67	43	4.44	55.12	8	199	14	2.669321	alpha	93.98285
1ajs	3418.66	666	192	63	39	10.26	46.87	21	421	26	3.859076	alpha	76.05085
1aor	1231.86	252	69	61	33	-2.79	42.64	9	182	10	4.726081	alpha	70.59203
1afw	2400	502	137	64	39	1.67	47.205	14	290	24	3.446717	alpha	77.30625
1ade	2750.36	561	151	61	23	-1.28	46.56	21	319	16	5.384568	alpha	65.93355
1b3a	745.96	171	45	61	37	4.42	40.32	6	103	7	2.80511	beta	83.85704
1b5e	2578.03	506	131	61	37	9.41	50.39	12	257	17	2.390488	beta	80.13037
1b8j	3789.715	783	205	63	40	-1.43	53.91	16	497	32	2.145378	coil	83.48623
1b67	1614.43	323	84	68	44	0.28	43.37	4	120	5	1.492632	alpha	80.00502
1bam	744.9	152	36	67	39	2	39.76	4	86	2	4.073191	alpha	74.57377
1bbh	745.56	171	46	74	46	1.4	43.415	6	70	0	9.338779	alpha	86.36126
1b8a	4284.65	900	230	67	50	18.46	84.935	28	588	28	2.451309	beta	91.52311
1bd0	3074.255	645	169	64	30	7.06	47.06	27	385	14	4.074034	coil	63.89467
1bif	898.1	198	56	59	22	-0.38	29.27	10	121	8	16.90318	alpha	65.65973
1biq	3017.62	606	147	66	37	4.79	49.86	14	378	23	1.823043	alpha	84.81303
1bis	1448.34	320	80	66	28	2.73	45.08	10	126	5	3.621215	alpha	81.09456
1bjw	2924.455	632	151	66	42	2.2	53.655	18	451	14	3.795706	alpha	93.08675
1bkp	2190.655	449	116	63	33	-1.72	48.595	11	250	18	3.025807	beta	83.25501
1bmd	1593.475	329	82	63	45	-3.13	45.525	10	251	8	4.649034	alpha	78.68213
1bsr	1921.445	395	94	64	38	6.46	50.77	12	232	12	3.154657	alpha/bet	79.01371
1bsl	1918.615	421	110	68	46	13.53	53.28	11	243	2	3.314109	alpha	89.97193
1brw	1058.14	235	60	69	49	2.6	44.395	7	108	6	8.2482	alpha	87.89527
1buo	1971.35	378	98	68	37	9.52	42.25	8	230	14	2.908728	alpha	84.62475
1bxg	1041.19	216	52	63	31	2.73	39.455	6	90	3	4.012956	alpha/bet	70.93566
1bxk	1270.26	273	80	68	42	2.42	40.105	8	96	3	4.115591	alpha	72.21159
1cdc	3863.615	774	170	67	46	6.95	65.535	2	510	47	1.121618	beta	88.11515
1cg2	1304.885	259	78	66	39	1.52	40.45	6	120	7	4.640064	beta	78.51765
1chm	3280.135	698	178	63	34	12.45	46.48	26	430	17	3.725722	coil	80.62
1cmb	1813.015	323	77	72	42	8.19	54.245	6	157	13	2.809061	alpha	85.33879
1coz	1046.905	208	48	75	42	5.52	40.13	6	133	4	1.885911	alpha	90.02202
1cnz	2472.965	493	124	68	45	7.54	45.245	13	339	19	4.630624	alpha	84.97512
1csh	5056.92	994	234	65	43	9.6	66.25	18	603	28	2.927893	alpha	80.40428
1czj	828.48	160	40	73	26	2.78	36.95	6	105	4	4.09002	coil	78.75024
1ctt	1989.96	410	106	66	36	6.1	53.76	16	215	11	2.481266	coil	84.88211
1dor	2184.46	436	117	69	34	8.71	44.79	16	216	15	2.691397	alpha	74.78851
1daa	2288.515	478	125	68	47	0.83	56.55	16	233	12	3.38014	coil	83.96493
1dqs	1631.105	345	97	65	34	-3.15	45.27	12	160	10	4.908022	coil	70.00898
1dpg	2277.945	448	125	70	40	3.82	46.445	14	200	15	2.940971	alpha/bet	72.71027
1dxg	739.36	149	42	62	44	2.6	40	4	99	11	0.488422	beta	85.18043
1e98	761.07	166	42	67	46	2.4	38.77	6	79	0	4.563141	alpha	75.81826
1ebh	1786.145	394	107	59	42	-2.35	44.015	16	250	25	6.738115	alpha	79.18198
1fip	1611.665	318	76	75	39	7.36	49.045	6	125	9	2.427068	alpha	86.14011
1fro	3624.965	735	199	64	37	15.87	49.905	10	374	18	3.321031	beta	68.4499
1cvu	2424.31	522	139	64	30	-1.48	44.71	15	319	15	5.020088	alpha	79.26668
1gvp	907.68	176	48	73	34	8.42	41.93	6	59	2	4.465638	beta	71.04045
1hhp	1566.17	328	72	64	42	5.86	55.9	10	251	13	1.935135	beta	83.21127

1hjr	952.68	203	52	72	43	2.91	41.775	4	94	1	3.191911	alpha	78.1847
1hss	1097.135	215	62	67	26	-0.9	38.225	4	102	2	2.565546	alpha	57.0937
1f13	2538.31	570	166	64	27	-1.7	34.935	37	243	15	8.492312	coil	61.03273
1hxp	3375.625	710	174	67	46	14.05	49.23	22	405	16	3.416366	beta	85.01418
1imb	1649.4	324	89	67	35	6.09	46.26	9	164	8	4.743088	alpha	73.85716
1icw	985.39	211	57	63	38	-0.98	40.68	5	97	11	2.479729	alpha/bet	85.46971
1isa	898.83	189	46	65	26	1.58	39.69	10	115	4	4.370682	alpha	91.15851
1jhg	2151.95	436	106	73	33	9.04	54.98	4	161	4	3.124143	alpha	72.23634
1jsg	794.47	160	42	69	38	6.32	35.5	8	74	4	4.757889	beta	70.59675
1ivy	1583.875	350	97	65	23	5.61	42.97	18	170	8	5.508244	coil	56.00158
1kpf	1827.99	388	92	62	51	1.08	55.94	8	321	16	1.482568	alpha	95.22426
1lyn	949.175	183	47	71	32	8.36	37.44	4	107	1	4.49798	alpha	77.94611
1m6p	1006.61	217	68	64	20	-3.36	34.43	8	81	3	5.046016	beta	53.95983
1mkb	1540.62	333	89	66	34	5.81	42.81	11	188	18	2.637574	beta	83.75946
1mor	2536.78	538	138	61	38	0.72	44.71	18	334	11	3.477736	alpha	75.68374
1nox	2966.39	608	150	66	38	13.76	50.02	12	292	24	3.859236	alpha	76.29138
1opy	1047.55	228	60	62	56	4.12	48.63	10	118	6	1.704329	beta	82.3932
1pgt	1232.145	237	64	65	43	4.67	39.78	6	127	6	6.957379	alpha	77.73071
1nsy	2560.055	533	127	69	35	1.47	45.77	10	282	10	3.170584	alpha	79.7645
1qr2	1903.35	422	106	69	35	9.45	45.74	14	216	9	4.284026	alpha	78.22996
1rfb	2645.36	551	140	73	12	2.84	47.905	6	181	3	4.886386	alpha	43.84847
1smt	1962	392	99	68	44	9.63	48.985	7	157	3	2.007452	alpha	84.01325
1smn	858.495	171	54	60	35	1.54	40.685	8	110	5	4.002062	coil	84.0069
1utg	1484.96	270	80	74	39	8.2	38.9	4	83	2	1.477313	alpha	81.6426
1tcl	1505.18	300	80	66	34	6.61	43.525	13	153	12	3.289387	alpha	70.78589
1rpo	1403.24	256	70	70	38	3.46	40.94	2	138	6	0.877875	alpha	70.48972
1uby	1766.5	360	100	64	18	-8.84	36.92	14	174	9	5.785168	alpha	57.58053
1vfr	3405.75	672	180	66	38	8.59	50.61	20	370	28	3.111649	alpha	81.24466
2tgi	1242.33	248	74	66	31	14.32	35.74	10	72	2	3.159483	beta	73.70022
1qfh	2284.815	486	118	62	34	-7.12	51.17	11	298	24	3.328002	beta	76.70862
2ccy	806.62	164	40	66	49	0.6	41.085	4	55	0	9.096291	alpha	83.05398
2mcg	1621.465	347	106	66	23	-2.97	33.495	15	146	2	6.138196	beta	53.92469
5csm	1935.32	406	114	67	30	3.06	37.25	20	182	4	4.167915	alpha	64.0292
4cha	1017.335	209	65	64	19	4.69	31.215	16	119	1	7.583785	coil	50.63573
2lig	1613.56	337	87	66	31	8.93	36.78	9	197	12	5.289775	alpha	76.22679
2tct	2662.59	546	126	69	33	7.6	47.41	14	247	11	2.666952	alpha	76.87289
9wga	2159.11	448	130	63	29	8.34	49.31	22	299	22	2.813659	coil	73.83691
2arc	805.64	177	39	67	37	2.74	40.85	6	108	1	3.338799	alpha	76.0532
2ohx	1713.145	355	97	64	43	5.96	45.24	8	208	9	4.657224	coil	84.26257
1vok	1579.42	311	93	75	24	6.32	33.78	14	138	3	4.228843	beta	73.71693
3sdh	885.955	178	53	63	35	-3.45	34.74	6	70	5	4.708478	alpha	76.93167
3ssi	866.14	177	62	64	40	0.38	39.365	6	62	2	3.771908	beta	79.79195
2ilk	4556.49	892	192	74	48	18.86	64.03	6	395	10	1.511771	alpha	81.85006
2spc	2509.385	479	115	70	38	8.11	42.585	4	298	6	2.048358	alpha	83.36565
5rub	2828.91	599	168	65	41	-0.9	52.655	18	330	20	3.421636	alpha	80.5874
3grs	3301.45	662	174	69	44	9.48	54.05	14	316	11	3.656687	alpha	82.18298
3dap	2631.01	544	141	68	43	6.76	52.47	10	225	16	3.34933	alpha	83.36228
2hdh	1518.875	311	80	73	27	5.64	43.42	6	127	4	3.155296	alpha	70.50021
1tox	3719.9	748	238	61	24	-2.42	45.55	30	320	8	5.863765	beta	65.3738
1r2f	1714.785	370	99	67	35	6.01	46.67	10	184	6	2.755447	alpha	67.86128
4kbp	1472.54	286	85	65	19	0.95	35.075	14	109	2	5.658841	coil	61.17355
1nse	2720.58	559	146	65	29	7.43	47.425	12	268	15	4.998151	alpha	79.81497
1qhi	1669.87	333	98	73	39	9.34	43.36	12	127	4	4.846635	alpha	76.52212
2nac	3752.34	765	196	66	49	7.84	58.9	16	473	19	2.487848	alpha	84.12164
12as	1879.005	368	102	72	26	6.77	37.565	16	149	2	4.941243	coil	64.38381
1ses	2218.01	441	118	69	51	12.42	49.565	14	258	13	3.26086	alpha/bet	90.25388
8prk	918.6	222	46	72	34	4.26	44.18	10	145	2	7.116667	coil	81.43098
1sox	1402.77	296	93	62	31	-0.79	39.835	16	217	8	8.281293	coil	64.94472
1trk	4467.455	920	234	63	34	-3.69	45.765	28	598	25	3.031675	alpha	85.36549
2sqc	807.575	155	46	68	26	0.1	36.29	8	77	1	12.75903	alpha	64.32591

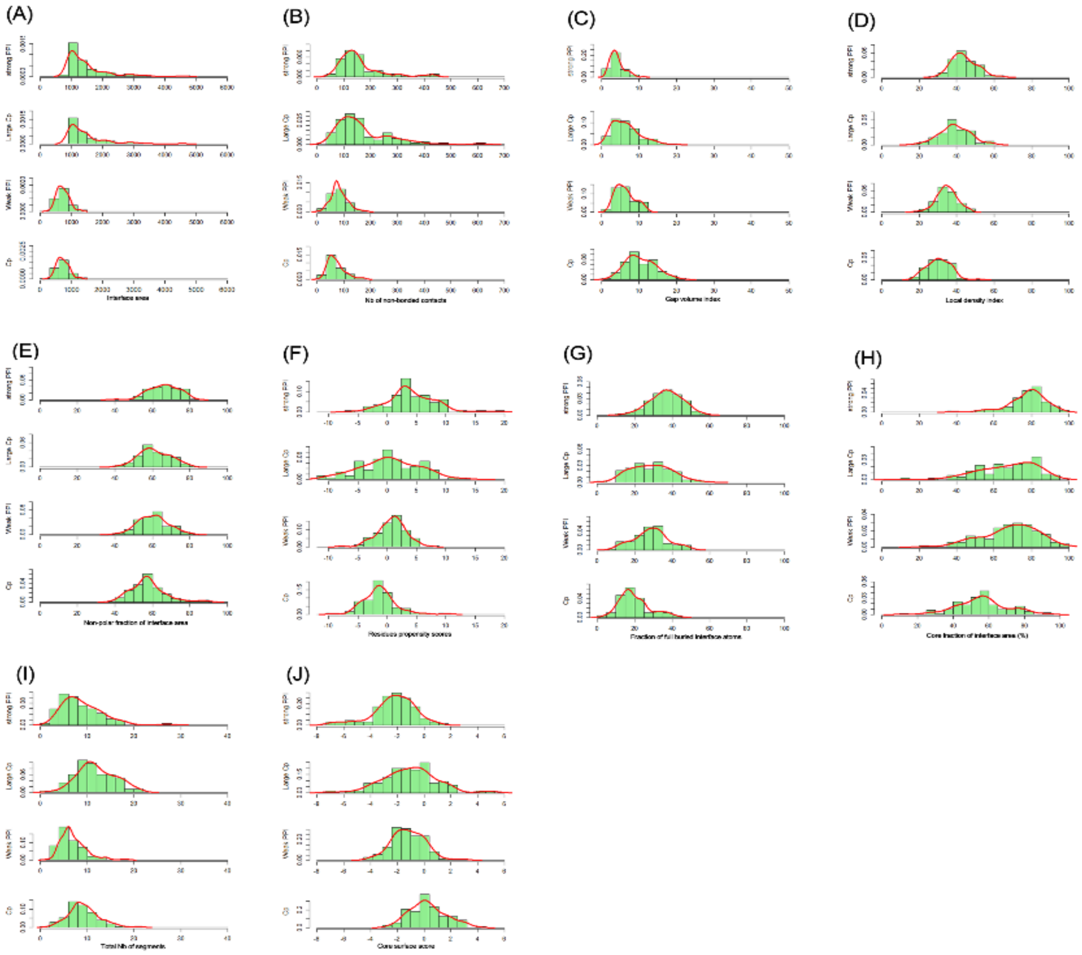

Supplementary Figure 1. The histograms of four types of PPI: (A) interface area; (B)number of non-bonded contacts; (C) gap volume index; (D) local density index; (E) non-polar area fraction; (F) residue propensity score; (G) fully buried atoms fraction; (H) core area fraction; (I) number of segments; (J) Core surface score
